# Rapid extraction-free detection of the R132H isocitrate dehydrogenase mutation in glioma using colorimetric peptide nucleic acid-loop mediated isothermal amplification (CPNA-LAMP)

**DOI:** 10.1371/journal.pone.0291666

**Published:** 2023-09-21

**Authors:** Kristian A. Choate, Edward J. Raack, Veronica F. Line, Matthew J. Jennings, Robert J. Belton, Robert J. Winn, Paul B. Mann

**Affiliations:** 1 Department of Biology, Northern Michigan University, Marquette, Michigan, United States of America; 2 Upper Michigan Brain Tumor Center, Marquette, Michigan, United States of America; 3 Northern Michigan University, Marquette, Michigan, United States of America; 4 School of Clinical Sciences, Northern Michigan University, Marquette, Michigan, United States of America; King Faisal Specialist Hospital and Research Center, SAUDI ARABIA

## Abstract

The R132H isocitrate dehydrogenase one (*IDH1*) mutation is a prognostic biomarker present in a subset of gliomas and is associated with heightened survival when paired with aggressive surgical resection. In this study, we establish proof-of-principle for rapid colorimetric detection of the *IDH1*-R132H mutation in tumor samples in under 1 hour without the need for a nucleic acid extraction. Colorimetric peptide nucleic acid loop-mediated isothermal amplification (CPNA-LAMP) utilizes 4 conventional LAMP primers, a blocking PNA probe complementary to the wild-type sequence, and a self-annealing loop primer complementary to the single nucleotide variant to only amplify the DNA sequence containing the mutation. This assay was evaluated using *IDH1*-WT or *IDH1-*R132H mutant synthetic DNA, wild-type or *IDH1*-R132H mutant U87MG cell lysates, and tumor lysates from archived patient samples in which the *IDH1* status was previously determined using immunohistochemistry (IHC). Reactions were performed using a hot water bath and visually interpreted as positive by a pink-to-yellow color change. Results were subsequently verified using agarose gel electrophoresis. CPNA-LAMP successfully detected the R132H single nucleotide variant, and results from tumor lysates yielded 100% concordance with IHC results, including instances when the single nucleotide variant was limited to a portion of the tumor. Importantly, when testing the tumor lysates, there were no false positive or false negative results.

## Introduction

Point mutations impacting the catalytic site of the isocitrate dehydrogenase enzyme in gliomas garner attention for their association with prolonged overall survival and improved prognosis [1]. The presence of an *IDH1* mutation is associated with a less aggressive phenotype, where *IDH1* mutant cells are more vulnerable to death [2] in comparison to those with the wildtype enzyme. Furthermore, the presence of an *IDH1* mutation enhances the radio-sensitization of malignant tissue [3,4]. The World Health Organization (WHO) considers the IDH status of certain tumor types to be sufficient for a complete diagnosis. The most recently released classification of tumors of the central nervous system (CNS5) lists IDH mutant astrocytoma and oligodendroglioma as a separate category from their wild-type counterparts [5]. *IDH1* mutations are observed in approximately 34% of glioma tumors [6].

Current treatment methods for patients with glioma include surgical resection, radiation therapy, and chemotherapy with the use of temozolomide (TMZ), yet the longterm survival rate remains poor. Studies indicate brain tumors harboring the *IDH1* mutation offer a median overall survival of 3.8 years compared to 1 year for patients with wild-type *IDH1* [7]. The difference in survival benefit is associated with the maximal surgical resection of tumors with the *IDH1* mutation [8–11]. This suggests that surgeons may decide to alter surgical plans intraoperatively and optimally resect tumor tissue to maximize the benefit of an *IDH1-R132* mutation. Real time knowledge of *IDH1* mutational status at the time of surgery would be ideal but is not currently practical. The presence of an *IDH1* mutation has traditionally been identified post-operatively via immunohistochemistry, Sanger sequencing or PCR [12,13]. These technologies are time-consuming and incapable of providing a real time result to a neurosurgery team. Another challenge for detecting *IDH1* mutations is that they are heterozygous at the cellular level [14] and heterogeneous at the tumor level [15]; thus, the mutation is present at relatively low copy number in clinical samples. To date, no published molecular diagnostic technique has demonstrated the ability to detect the *IDH1* genotype during the surgical window.

**Fig 1:**
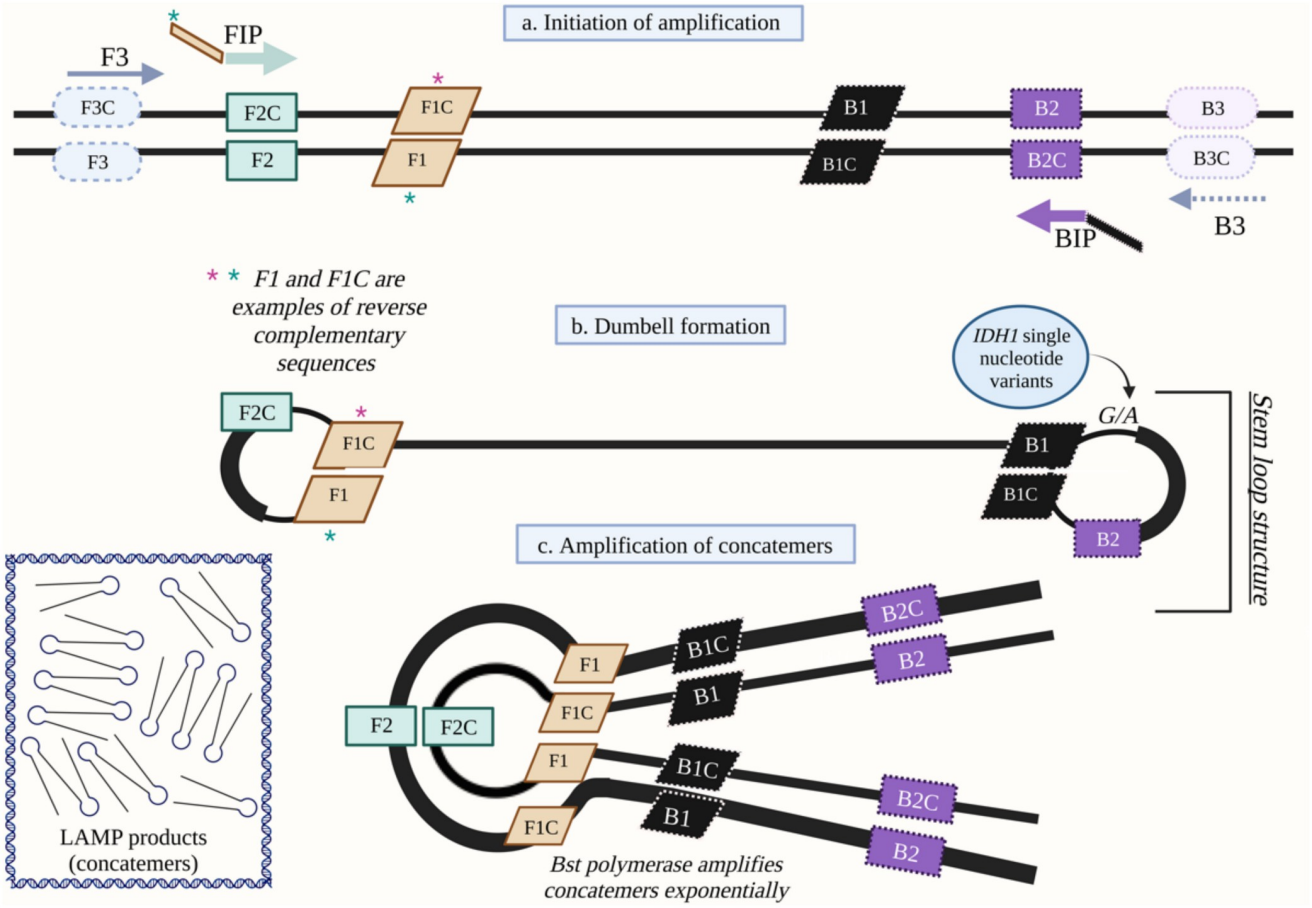
Mechanism of DNA amplification via loop-mediated isothermal amplification (LAMP). (a) Amplification initiates by strand invasion via inner primers (FIP & BIP). FIP consists of F1C and F2 sequences, while BIP consists of B1C and B2. Bst polymerase extends these primers and continues displacement of DNA. (b) Another set of primers (outer primers, F3 & B3) displace the initial product, which then form self-hybridizing loop structures or ’dumbbells’ due to reverse complementary sequences in FIP and BIP. The dumbbell structure contains the IDH1-R132H single nucleotide variant (SNV) (guanine→adenine) (c) The dumbbell structures have multiple sites for synthesis to initiate, resulting in rapid/exponential amplification and the production of concatemers.

Loop-mediated isothermal amplification (LAMP) is a technique capable of rapidly amplifying and detecting target DNA sequences [16]. LAMP employs four primers: forward/backward inner primers (FIP, BIP) and forward/backward outer primers (F3, B3) that recognize six distinct regions in the target sequence (Fig 1) and that greatly enhance specificity and sensitivity. Amplification from F3 and B3 result in strand displacement, generating single-stranded DNA that can serve as another template. FIP and BIP each contain two target sequences specific to four different regions in the template DNA. *Bst* polymerase will initiate amplification from these primers followed by the subsequent displacement of the complementary strand. Since the 5’ ends of FIP and BIP are the reverse complement to sequences further inwards on the template, stem-loop structures form, enabling exponential amplification of the target (Fig 1 and S1 Fig).

**Fig 2:**
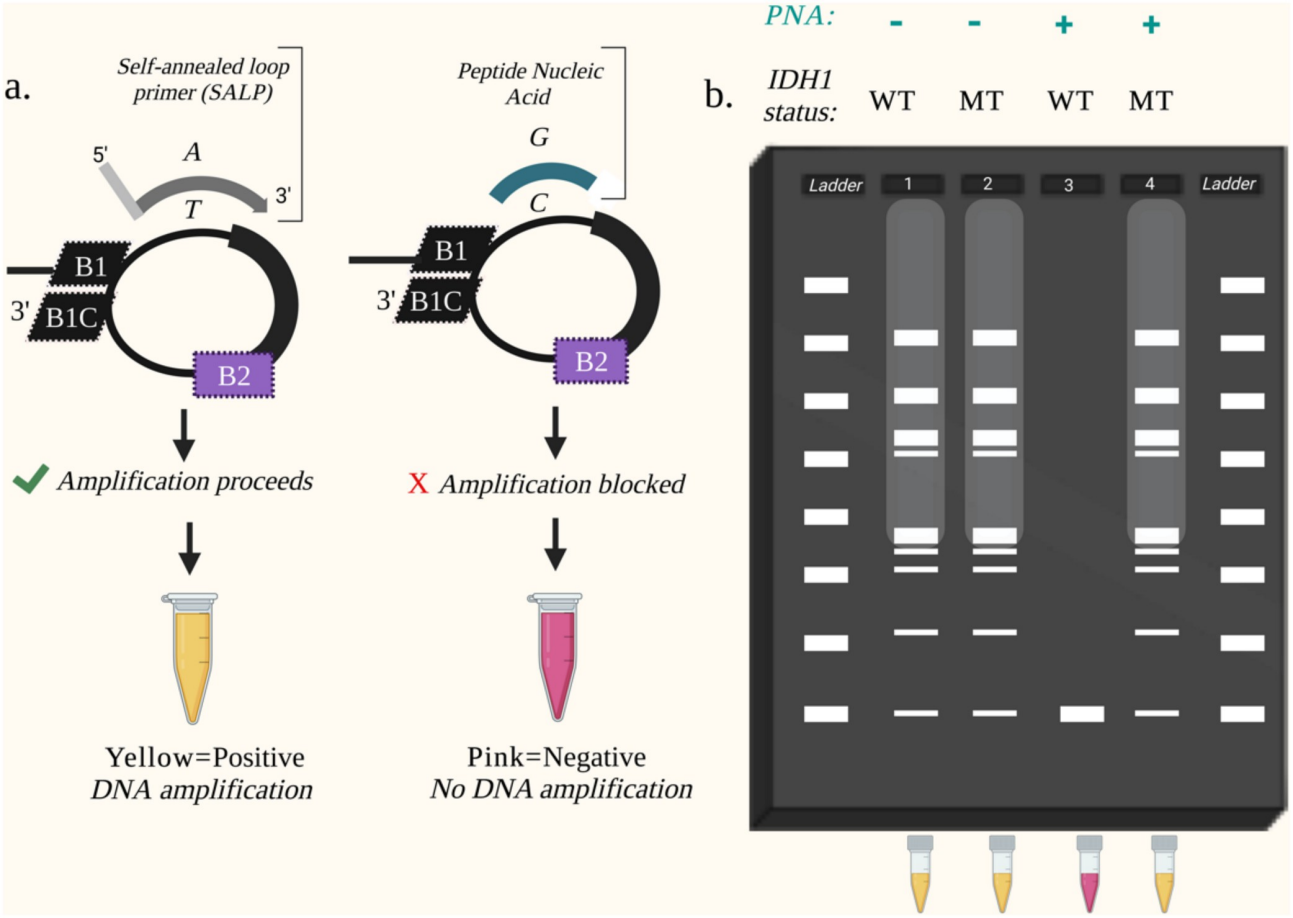
General properties of IDH1-R132H specific CPNA-LAMP. (a) The self-annealing loop primer preferentially enhances the amplification of mutant DNA. In the absence of PNA, the SALP will also assist in the amplification of WT IDH1 DNA. The peptide nucleic acid blocks the amplification of IDH1 wild-type DNA. (b) An example of a gel electrophoresis result of CPNA-LAMP samples. Lane 1: In the absence of the peptide nucleic acid, WT DNA amplifies. Lanes 2&4: IDH1-R132H mutant (MT) DNA amplifies rapidly with or without PNA presence. Lane 3: If only wild-type DNA is present, amplification is suppressed by the complementary peptide nucleic acid.

To specifically detect the *IDH1*-R132H mutation, we modified conventional LAMP to include a self-annealing loop primer (SALP) complementary to the R132H variant and a peptide nucleic acid (PNA) blocking probe complementary to the wildtype sequence (Fig 2 and S1 Fig). The combination of the SALP and PNA within this LAMP reaction are designed to allow discrimination between wild-type and *IDH1* mutant genotypes. PNAs are molecules that display properties of both DNA and peptides. The chemical structure most closely represents a peptide, while the behavior mimics DNA in ways including adherence to Watson-Crick-Franklin base pairing. The neutrality of the pseudopeptide backbone offers a strong binding affinity between PNA and DNA due to a lack of charge repulsion, with higher stability and T_m_ [17] than naturally occurring DNA-DNA duplexes. The stability, mismatch discrimination and favorable hybridization displayed by PNAs make them a desirable tool for diagnostic assays. Furthermore, the lack of a 3’-hydroxyl group on the PNA prevents *Bst* polymerase from extending the sequence. Peptide nucleic acid LAMP (PNA-LAMP) was previously shown to preferentially block amplification of wild-type sequences containing only a single nucleotide difference while permitting amplification of mutated sequences [18].

LAMP can be performed using any equipment that provides a constant set temperature, such as a water bath or heat block, omitting the need for a thermocycler. Positive LAMP reactions can be determined by various colorimetric techniques, thus facilitating visual detection methods, and further reducing the need for complex laboratory equipment [19]. Specific to this study, a pH-sensitive colorimetric dye allows for a vibrant pink-to-yellow color change indicating a positive result upon amplification of the target sequence. Gel electrophoresis to confirm positive LAMP reactions yields a laddering pattern of various sizes due to the presence of multiform concatemers rather than a single band of a specific size as typically seen in PCR (Fig 2), [20].

In this study, we investigated the use of a nucleic acid extraction free CPNA-LAMP assay to specifically and rapidly amplify *IDH1*-R132H mutant DNA in patient-derived tumor samples. The assay was evaluated using samples of increasing complexity to assess the feasibility of directly analyzing tumor lysates. Here, we demonstrate the specific detection of *IDH1*-R132H from crude tumor homogenates in under an hour using CPNA-LAMP with minimal preanalytical processing and without the use of complex equipment.

## Materials and methods

### Primer design

LAMP primers were designed using Eiken’s PrimerExplorer primer design tool. The 16-mer PNA (Panagene) contains one lysine residue on each end to enhance solubility and is complementary to the WT allele between the B1 and B2 regions of the LAMP amplicon. The SALP is complementary to the *IDH1-*R132H sequence located between the B1 and B2 sites on the LAMP dumbbell structure. Oligonucleotides were obtained from Integrated DNA Technologies (IDT).

### Colorimetric PNA-LAMP

CPNA-LAMP was performed using WarmStart^®^ Colorimetric LAMP Master Mix (New England Biolabs, NEB), custom LAMP primers (IDT, Panagene), and 1M betaine (SigmaAldrich). LAMP master mix contains Bst polymerase, an enzyme derived from the large fragment of *Bacillus stearothermophilus* DNA Polymerase I. LAMP reactions were performed in nuclease free PCR tubes at a 25μL reaction volume. Primer concentrations were 0.2 μM F3, 0.2 μM B3, 1.6 μM FIP, 1.6 μM BIP, 0.8 μM R132H SALP, and 0.25 μM PNA. Reactions were incubated at 65°C for 45–60 minutes, then denatured at 95°C for 2 minutes. Amplification was confirmed via 4% agarose gel electrophoresis at 100 volts for 1 hour then imaged with a BioRad ChemiDoc MP^™^ Imaging System.

### Samples

**Human genomic DNA (hgDNA)** was extracted from telomerase-immortalized human endometrial stromal cells (ATCC, CRL-4003) and purified using the QIAamp DNA Mini Kit protocol (Qiagen).

**Bacterial *Pseudomonas aeruginosa*** DNA was extracted from confluent inoculated culture and purified using the QIAamp DNA Mini Kit protocol (Qiagen) for use as a negative control.

**Synthetic WT and *IDH1*-R132H DNA** (653 BP) containing the region with the single nucleotide variant were purchased from IDT.

***IDH1* wild-type U87MG cells and CRISPR edited *IDH1* isogenic U87HTB-14IG**^**™**^ **U87MG cells** (ATCC, Manassas, VA, USA) were cultured in standard conditions with Eagle’s Minimum Essential Medium (EMEM) (Lonza, Portsmouth, NH, USA), 10% fetal bovine serum (Atlanta Biologicals, Norcross, GA, USA) and 1% penicillin/streptomycin/amphotericin B (PSA) (Lonza, Portsmouth, NH, USA). Cells were harvested at a concentration of 10^5^ cells per μL and the samples lysed by vortex in physiological saline in the presence of 10% proteinase K (>600 mAU/mL Qiagen, Hilden, Germany) per volume. The samples were incubated at 56°C for 10 minutes, then immediately denatured at 95°C for 15 minutes. Following denaturation, samples were vortexed and then stored at -80°C until use.

**Archived Tumor Samples** with known *IDH1* mutational status were provided by Advocate Aurora Research Institute, LLC, Milwaukee, WI. Tumor lysates were created by homogenizing approximately 0.1 gram of tumor in 500 μL of physiological saline, then diluting to approximately 10^4^ cells per μL in molecular grade water. Lysates were incubated in the presence of 10% proteinase K (>600 mAU/mL Qiagen, Hilden, Germany) per volume at 56°C for 10 minutes then 95° C for 15 minutes. Alternatively, lysates were incubated with 0.4 M NaOH for 5 minutes followed by a 1:100 dilution into molecular grade water. Cells obtained from the post-surgical tumor filters were diluted to approximately 10^4^ cells per μL. A final 1:20 dilution was prepared for each sample, and 2 μL of sample was used per 25 μL reaction.

## Results

### Detection of *IDH1* using colorimetric LAMP

**Fig 3:**
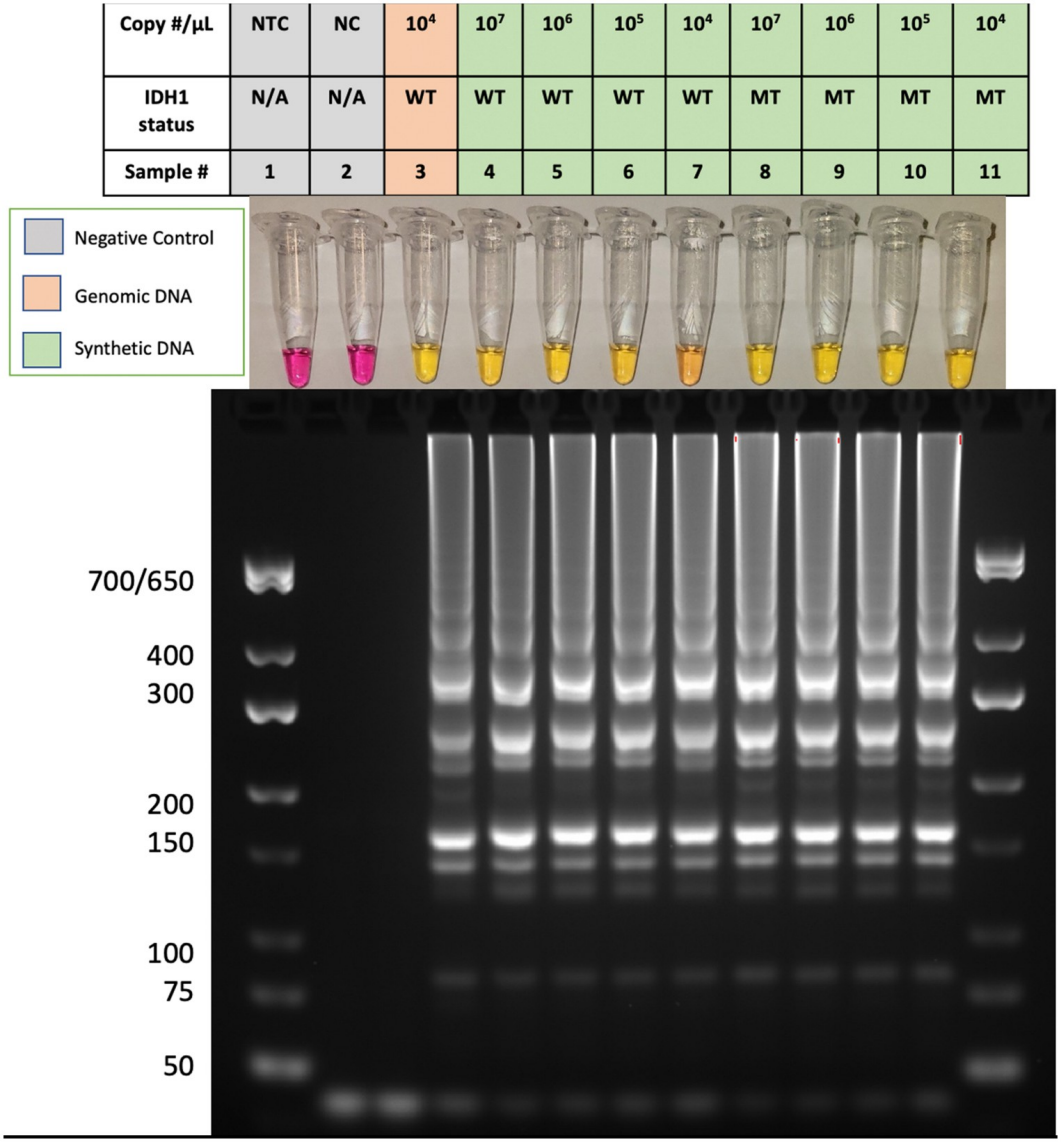
Detection of IDH1 using colorimetric LAMP. **Pink colorimetric results denote a negative result, while yellow indicates a positive result**. (1) Non-template control, Pseudomonas Aeruginosa gDNA (NTC), (2) Negative template control (NC), (3) hgDNA, (4–11) varying concentrations of synthetic wildtype (WT) and mutant (MT) DNA. **Results are representative of 3 or more experimental runs performed by >3 technicians**.

To determine the feasibility of amplifying the region of the *IDH1* gene harboring the R132H mutation, we applied decreasing concentrations of synthetic wild type (WT) and *IDH1-*R132H synthetic DNA to the LAMP assay in the absence of PNA and incubated at 65°C for 55 minutes. Positive LAMP results were detected by observing colorimetric changes (from pink to yellow) in tubes containing WT and R132H synthetic DNA at copy numbers ranging from 5.0X10^7^ to 5.0X10^4^ per reaction. Gel electrophoresis of the samples revealed a laddering pattern typical of a positive LAMP reaction due to the formation of concatemers which are multimers of the initial LAMP template. A robust color change was observed in tubes containing either the WT or the R132H sequence. Furthermore, LAMP was able to amplify hgDNA containing 5.0X10^4^ copies of the *IDH1* gene (Fig 3). This result demonstrates the LAMP assay’s ability to amplify wildtype or mutant IDH1 gene sequences and *IDH1* within hgDNA.

### Specific detection of *IDH1-*R132H using CPNA-LAMP

**Fig 4:**
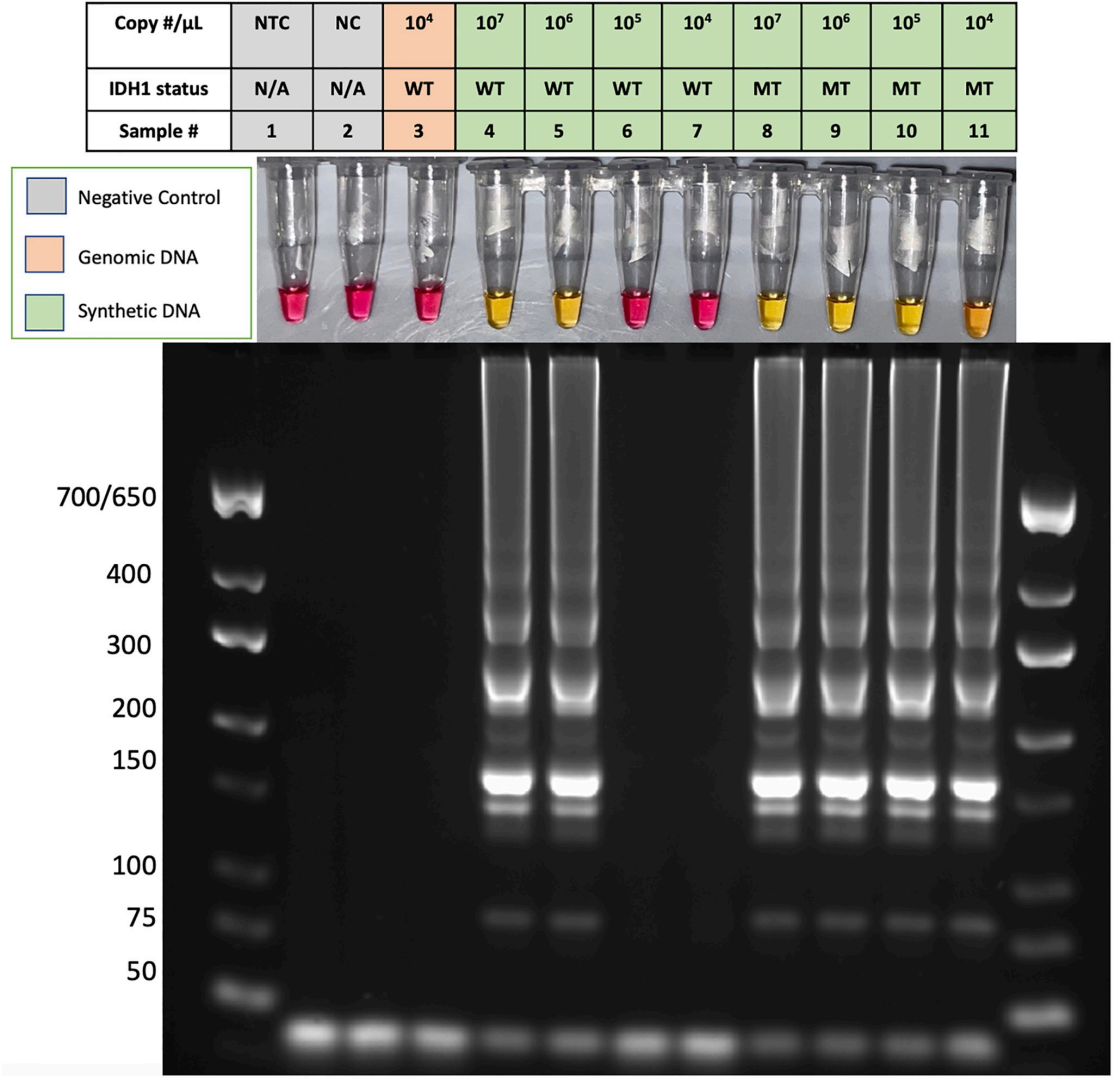
Specific detection of IDH1-R132H MUT using CPNA-LAMP. Pink colorimetric results denote a negative result, while yellow indicates a positive result. All reactions contain PNA. (1) Non-template control (NTC), (2) Negative template control (NC), (3) hgDNA, (4–11) varying concentrations of synthetic wildtype (WT) and mutant (MT) DNA. Results are representative of 3 or more experimental runs performed by >3 technicians.

We next tested the ability of the PNA to prevent amplification of the wild-type sequence while allowing specific amplification of the *IDH1-*R132H sequence. For this, decreasing copy numbers of synthetic WT and R132H mutant *IDH1* sequences were analyzed. Reactions containing the R132H synthetic DNA from 5.0X10^7^ to 5.0X10^4^ copies per reaction developed a robust color change and the gel electrophoresis patterns confirm positive LAMP reactions (Fig 4). Wildtype *IDH1* synthetic DNA or human genomic DNA did not amplify at copy numbers at or below 5.0X10^5^. A timecourse analysis demonstrated unambiguous colorimetric detection of the *IDH1*-R132H mutation at 55 minutes (S4 Fig). A more detailed analysis determined that the PNA suppresses amplification of the wildtype *IDH1* sequence at copy numbers below 5.0X10^5^ (S5 Fig), allowing for specific detection of the R132H mutant at defined copy numbers. Collectively, these findings demonstrate that the CPNA-LAMP method specifically detects the *IDH1-*R132H mutation. To assure reliable suppression of the wildtype sequence, subsequent CPNA-LAMP reactions were performed using ≤ 5.0X10^4^ copies of *IDH1*.

### Detection of *IDH1*-R132H in the presence of wildtype DNA

**Fig 5:**
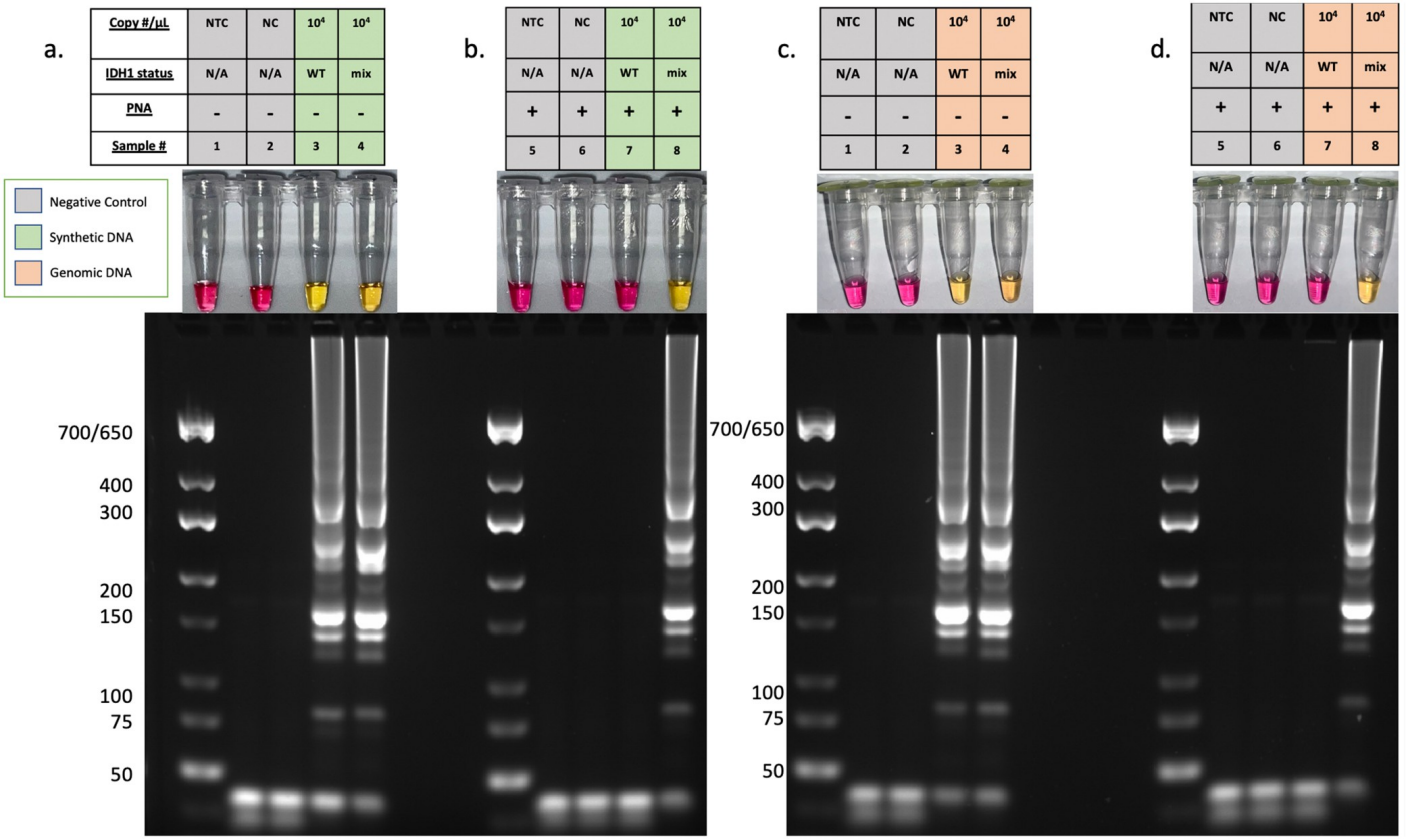
Detection of IDH1-R132H in a background of WT synthetic DNA (A, B) or hgDNA (C,D). Reactions in A and C amplify all IDH1 sequences due to the absence of PNA, while samples in B and D are specific to IDH1-R132H due to the inclusion of PNA. Results are representative of 3 or more experimental runs performed by >3 technicians. (1) NTC, (2) NC, (3) WT at 5.0X10^4^ copies, (4) WT and R132H mutant at 2.5X10^4^ each (total: 5.0X 10^4^), (5) NTC, (6) NC, (7) WT at 5.0X10^4^ copies, (8) WT and R132H mutant at 2.5X10^4^ each (total: 5.0X 10^4^).

Due to tumor heterogeneity and *IDH1* allele heterozygosity, the ability to detect the mutant sequence in a background of wild-type DNA is required for this assay to be useful in the detection of *IDH1* SNVs in tumor samples. Next, we sought to determine its ability to detect the mutation in reactions containing equal copy numbers of R132H mutant and WT sequences (Fig 5A and 5B). Additionally, R132H synthetic DNA was spiked into a background of hgDNA to further increase the complexity of the sample (Fig 5B). Without PNA, the LAMP assay amplified wild type and mutant *IDH1* (Fig 5A and 5C). However, the addition of PNA to the assay suppressed amplification of the wildtype *IDH1*, permitting specific detection of the R132H SNV (Fig 5B and 5D). Gel electrophoresis confirmed colorimetric results and further demonstrated the assay’s high specificity. Consistent with previous results, the CPNA-LAMP assay specifically detected the R132H sequence in a background of excess hgDNA.

### Amplification and detection of *IDH1*-R132H without DNA extraction

**Fig 6:**
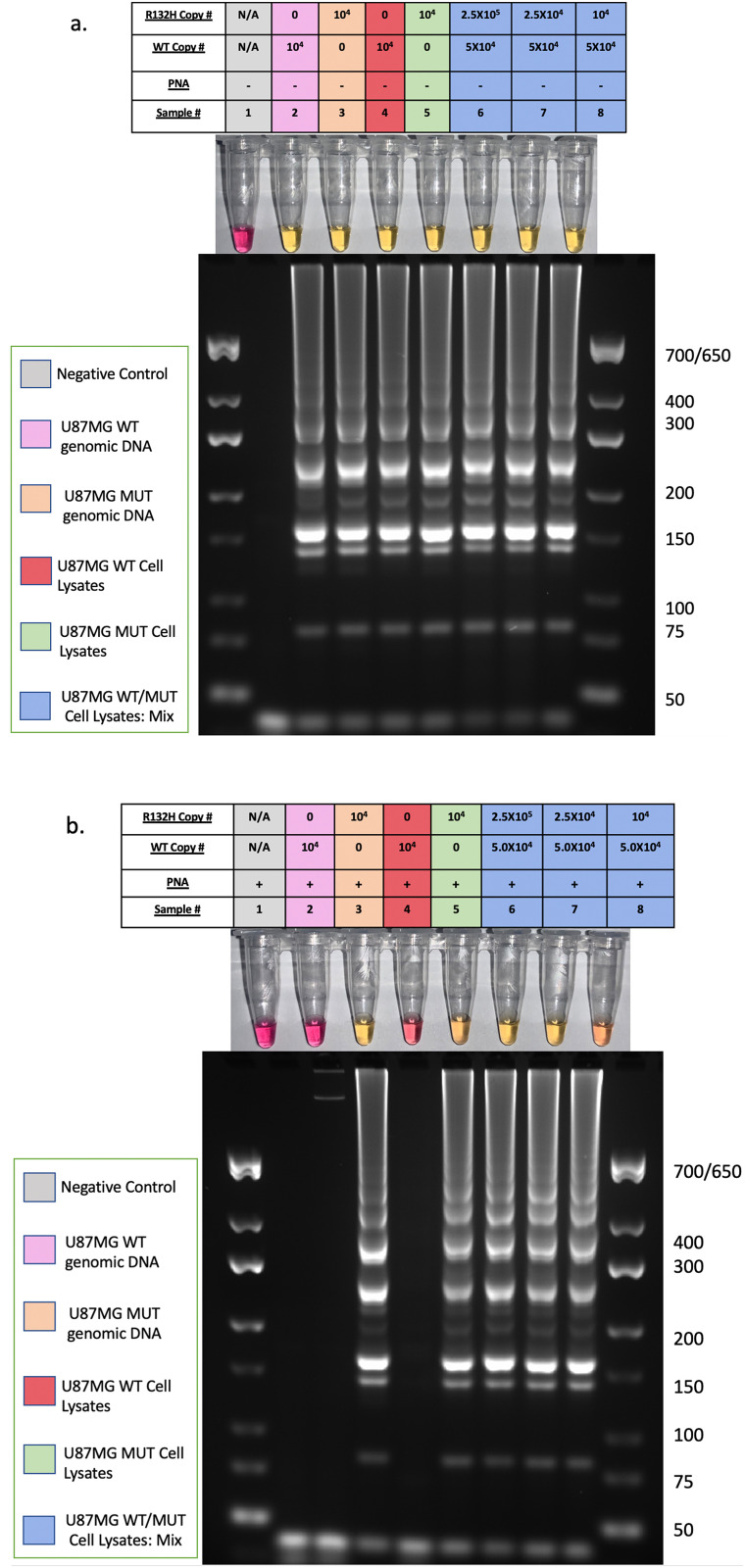
Detection of R132H with CPNA-LAMP does not require a DNA extraction. a. reactions were performed without PNA. b. reactions were performed with PNA. (1) NC, (2) purified WT Genomic DNA at 5.0X10^4^ copies, (3) purified R132H MT Genomic DNA at 5.0X10^4^ copies, (4) U87MG cell lysate containing 5.0X10^4^ copies of IDH1, (5) U87MG IDH1-R132H cell lysate containing 2.5X10^4^ copies of WT and 2.5X10^4^ copies of R132H MT. The following U87MG IDH1-R132H cell lysates are spiked into a background of IDH1 wildtype U87MG lysate at 5x10^4^ copies. (6) 1.0X10^5^ copies of IDH1-R132H, (7) 2.5X10^4^ copies of IDH1-R132H, (8) 1.0X10^4^ copies of IDH1-R132H. Results are representative of 3 or more experimental runs performed by >3 technicians.

We next sought to determine if CPNA-LAMP could specifically detect the R132H mutation in crude lysates from *IDH1*-R132H U87MG cells. LAMP amplified *IDH1* in all lysates (Fig 6A) and CPNA-LAMP specifically detected the R132H mutation in lysates possessing the SNV (Fig 6B) thus demonstrating the efficacy of the assay in the absence of a nucleic acid extraction step. Since tumors are heterogenous, we next evaluated the assay’s ability to detect low copy numbers of the mutant sequence in a background of excess wildtype *IDH1*. As few as 1.0X10^4^ copies of *IDH1-R132H* in a background of 5.0X10^4^ WT *IDH1* copies per reaction were sufficient to detect the mutant. This indicates that the CPNA-LAMP method can specifically detect the mutation in a sample containing an excess of wildtype *IDH1*. Importantly, the CPNA-LAMP method did not require a DNA extraction step to detect a comparatively low copy number of *IDH1*-R132H.

### Amplification of *IDH1* in patient-derived tumor lysates

**Fig 7:**
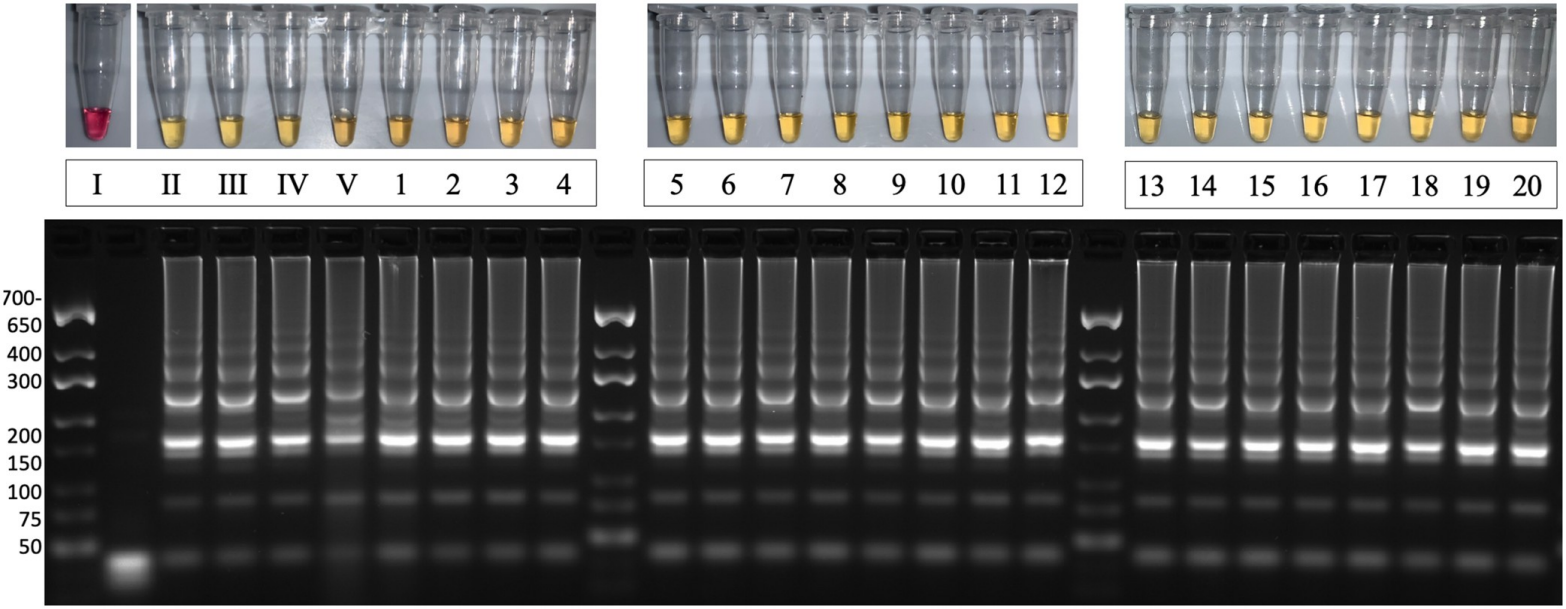
LAMP amplifies WT or MT IDH1 in patient-derived tumor samples without a nucleic acid extraction. Lane (I) NTC, (II) WT IDH1 DNA at 5x10^4^ copies (III) IDH1-R132H DNA at 5x10^4^ copies (IV) IDH1 WT U87MG lysate at 5x10^3^ cells (V) IDH1-R132H U87MG lysates at 5x10^3^ cells (1–20) archived patient tumor lysates. Results are representative of 3 or more experimental runs performed by >3 technicians.

We next sought to determine if LAMP could amplify *IDH1* in patient tumors. Lysates were prepared using tumor tissue from 20 archived frozen samples. Prepared lysates were diluted in physiological saline then added directly to the LAMP reaction. We demonstrated amplification of the *IDH1* sequence in all samples regardless of the mutational status of the *IDH1* gene (Fig 7). This provides proof of concept that LAMP is capable of amplifying the *IDH1* sequence from tumor lysates. Thus, we hypothesized that the *IDH1* LAMP assay modified with the PNA and SALP may be used to detect the R132H sequence.

### Specific detection of *IDH1*-R132H in patient-derived tumor lysates

**Fig 8:**
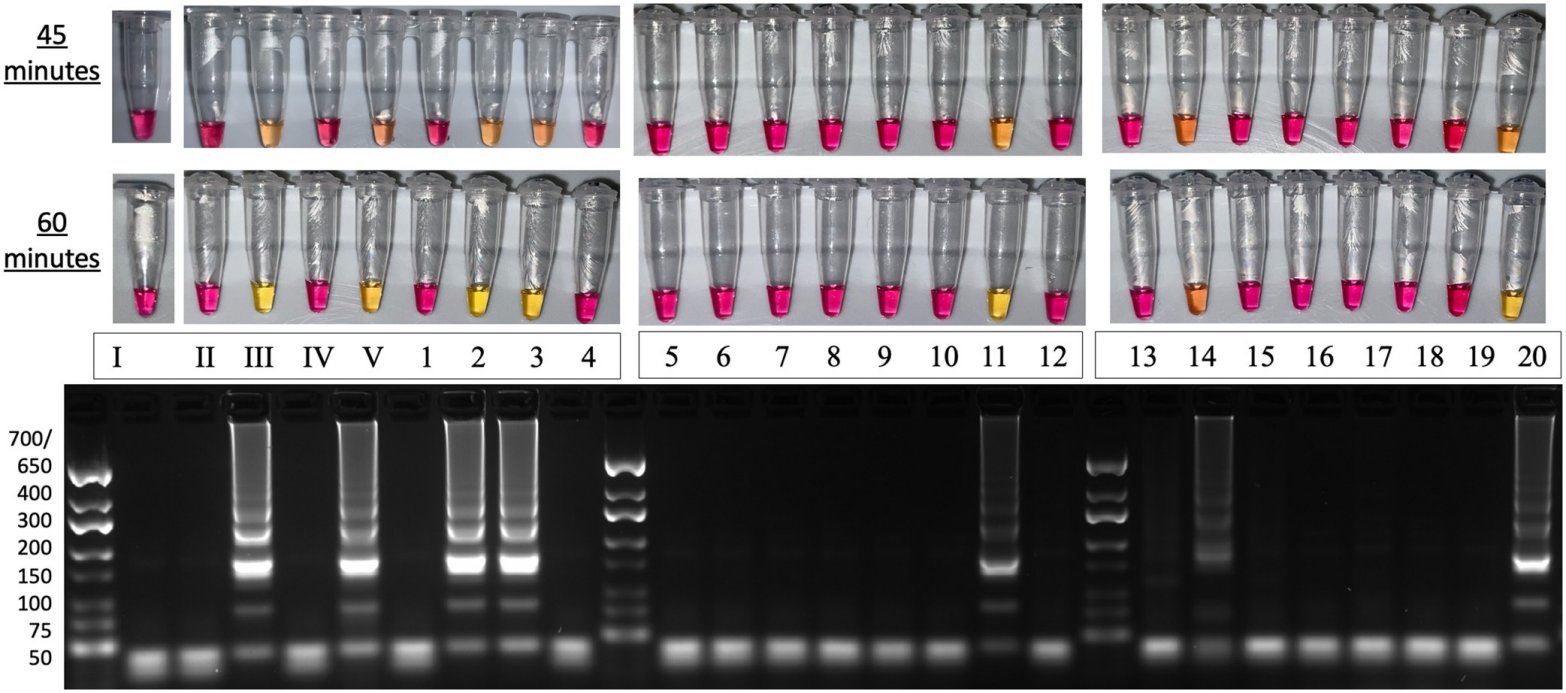
CPNA-LAMP specifically detects IDH1-R132H in patient-derived tumor samples. Lane (I) NTC, (II) WT IDH1 DNA at 5x10^4^ copies (III) IDH1-R132H DNA at 5x10^4^ copies (IV) IDH1 WT U87MG lysate at 5x10^3^ cells (V) IDH1-R132H U87MG lysates at 5x10^3^ cells (1–20) archived patient tumor lysates. Sample number corresponds with information found in Table 1. Gel electrophoresis was performed on the samples incubated for 60-minutes. Results are representative of 3 or more experimental runs performed by >3 technicians.

Since we demonstrated that PNA was able to suppress amplification of WT *IDH1* (Figs 4, 5B, 5D and 6B), and LAMP amplified *IDH1* in tumor lysates (Fig 7), we next sought to evaluate if CPNA-LAMP can specifically detect *IDH1*-R132H using tumor lysates. Of the 20 lysates tested, the five reactions containing the *IDH1* mutation amplified, while the remaining 15 reactions only containing WT *IDH1* did not amplify (Fig 8). Importantly, these results show 100% concordance with previously reported pathology results as shown in Table 1. Technicians preparing the reactions were blinded to previous *IDH1* pathology results and data are representative of multiple experiments. Samples containing the mutation (2, 3, 11, 14, and 20) yielded visibly positive results after approximately 45 minutes. These results demonstrate that CPNA-LAMP specifically detects the *IDH1*-R132H mutation in glioma tumor samples in less than one hour without the need for nucleic acid extraction.

**Table 1:** Comparison of IDH1-R132H PNA LAMP results with pathology report.

Sample #	Diagnosis	*IDH1* Pathology Report	CPNA-LAMP Result
**1**	Glioblastoma multiforme	Not Detected	Negative
**2**	Glioblastoma*	Positive in a subpopulation of the tumor	Positive
**3**	Glioblastoma*	Positive in a subpopulation of the tumor	Positive
**4**	Glioblastoma multiforme (WHO grade IV astrocytoma)	Negative	Negative
**5**	Glioblastoma multiforme (WHO grade IV)	Negative	Negative
**6**	Glioblastoma multiforme (WHO grade IV)	Negative	Negative
**7**	Glioblastoma multiforme	Negative	Negative
**8**	Glioblastoma multiforme (WHO grade IV)	Negative	Negative
**9**	Glioblastoma (WHO grade IV)	Negative	Negative
**10**	Glioblastoma multiforme	Negative	Negative
**11**	High Grade Glioma	Positive	Positive
**12**	Glioblastoma multiforme (WHO grade IV)	Not Detected	Negative
**13**	Glioblastoma multiforme	Negative	Negative
**14**	Anaplastic astrocytoma	Mutation Detected	Positive
**15**	Glioblastoma multiforme (WHO grade IV)	Negative	Negative
**16**	Glioblastoma multiforme (WHO grade IV)	Negative	Negative
**17**	Glioblastoma multiforme	Negative	Negative
**18**	Glioblastoma multiforme (WHO grade IV)	Negative	Negative
**19**	Glioblastoma multiforme	Negative	Negative
**20**	Anaplastic astrocytoma	Positive	Positive

* This pathology report was provided to us prior to the release of the CNA5 by the WHO. While samples 2 and 3 are listed as glioblastoma (GBM), new classifications place astrocytoma with IDH mutations in a separate category from GBM [5].

## Discussion

In this study, we have developed a CPNA-LAMP assay to specifically detect *IDH1-* R132H mutations rapidly in patient-derived glioma tumor lysates. To accomplish this, we initially demonstrated the ability of CPNA-LAMP to accurately detect the mutation using purified synthetic DNA (Fig 4). We subsequently tested the ability of the assay to specifically detect *IDH1*-R132H in increasingly complex matrices (Figs 5 and 6). Lastly, we evaluated the performance of the assay on patient-derived tumor lysates. CPNA-LAMP analysis of 20 archived tumor samples by technicians blinded to the mutational status showed 100% concordance with previous pathology reports (Fig 8) and Table 1. The CPNA-LAMP assay was found to be highly reproducable, as all data obtained were representative of a minimum of three runs by four different technicians. Notably, we utilized tumor lysates that were manually homogenized in physiological saline and exposed to a brief enzymatic digestion or subjected to NaOH lysis (S6 Fig).

Despite the accuracy and reproducibility of the CPNA-LAMP assay, there are limitations which should be addressed. While the PNA is necessary to suppress amplification of wildtype *IDH1*, it also reduces the rate of amplification of all *IDH1* sequences and has a limited capacity to suppress amplification in reactions containing >5.0X10^5^ copies of the wildtype sequence. Thus, the stoichiometry of the PNA with wildtype *IDH1* must be balanced to reliably inhibit wildtype amplification while allowing for rapid detection of the mutant. While it is feasible to estimate the approximate DNA copy number from a tumor sample based upon weight, it is not possible to determine the exact copy number. Despite this, we achieved reproducible and accurate results by utilizing tissue weight as a means of cell number estimation. While the initial tumor lysates for this study were prepared using a proteinase K digest and denaturation step, subsequent findings demonstrated a simple NaOH lysis step to be more rapid and provided identical results (S6 Fig). Additionally, the scope of this initial study was limited to 20 patient derived tumor samples. Thus, further optimization and testing of this method is warranted.

Knowledge of the *IDH1* mutational status for a particular glioma can enable neurosurgeons to make informed decisions regarding the extent of resection. However, current methodologies are unable to provide this information in real-time during surgery. Our work demonstrates the ability to rapidly detect the mutation when present at less than 20% in the sample (Fig 6B), which is a level of sensitivity comparable to Sanger Sequencing. Additionally, the use of a pH-based CPNA-LAMP method allowed for real-time visual detection of a positive result. These findings suggest that the method may be performed in close proximity to the surgery, providing rapid diagnostic support. Mutations to *IDH1* are thought to be tumorigenic [21] and the R132H single nucleotide variant is established as an important cancer biomarker. Recently, it was demonstrated that gliomas can lose this mutant allele in a portion of tumors [22]. Researchers thus suggested the importance of routine monitoring of the *IDH1* mutational status during therapy. This places an increased emphasis on the need to rapidly and accurately detect an *IDH1* variant.

Although the R132H mutation is the most prevalent, other R132 variants have been documented with a similar tumor phenotype [6]. Thus, being able to detect these additional variants is clinically relevant. Though the SALP in this study was designed to detect the R132H SNV, it will also amplify R132C DNA (S2 and S3 Figs), albeit at a slower rate. We suggest this difference in efficiency may be the result of a single nucleotide mismatch between the SALP and template. Therefore, the assay can be modified using different SALPs to detect the different R132 codon mutations. This hypothesis is supported by a PNA-LAMP method that was developed to specifically detect single nucleotide variants (SNVs) in the *KRAS* gene using DNA extracted from *KRAS* mutant and wildtype cell lines and formalin-fixed paraffin embedded samples [23].

Development and deployment of a real-time *IDH1* genotyping platform will enhance intraoperative diagnostic capabilities and inform extent of resection decisions. To this end, small footprint clinical analyzers have been developed to perform LAMP assays. The method demonstrated in this study requires minimal preanalytical processing and can thus be adapted to a similar device that could standardize sample input and optimize the limit of detection. The adaptation of this method to such a device would provide neurosurgeons with real-time knowledge of the *IDH1* status of tumors.

## Supporting information

S1 Fig An overview of IDH1-R132H specific PNA-LAMP primers utilized within this study. Forward inner primer (FIP) and backward inner primer (BIP) contain two target sequences specific to two different regions in the template DNA. Four thymine linkers provide flexibility for FIP to create secondary structures. The optional addition of loop primers that target dumbbell loops can further expedite amplification. The SALP contains self-complementary ends which form stem-loop structures until bound to the target sequence.
(TIF)

S2 Fig Amplification of IDH1-R132C using colorimetric LAMP. (1) Non-template control, (2) WT at 5.0X10^4^ copies, (3) IDH1-R132H at 5.0X10^4^ copies, (4) IDH1-R132C at 5.0X10^4^ copies, (5) WT at 5.0X10^5^ copies, (6) IDH1-R132H at 5.0X10^5^ copies, (4) IDH1-R132C at 5.0X10^5^ copies.
(TIF)

S3 Fig Amplification of IDH1-R132C using colorimetric LAMP. (1) Non-template control, (2) WT at 5.0X10^4^ copies, (3) IDH1-R132H at 5.0X10^4^ copies, (4) IDH1-R132C at 5.0X10^4^ copies, (5) WT at 5.0X10^5^ copies, (6) IDH1-R132H at 5.0X10^5^ copies, (4) IDH1-R132C at 5.0X10^5^ copies.
(TIF)

S4 Fig A time-lapse of CPNA-LAMP yields unambiguous detection of the IDH1 R132H mutation at 55 minutes. Lanes 1–4 do not contain PNA while lanes 5–8 contain PNA. Odd samples contain IDH1 wildtype synthetic DNA while evenly numbered samples contain IDH1-R132H mutant synthetic DNA. The presence of PNA in the reaction delays colorimetric changes by approximately 10 minutes. Positive results become visually interpretable at 45 minutes, then unambiguous by 55. Higher copy number of DNA results in an earlier and more vibrant colorimetric change. Importantly, at 55 minutes, when there is no colorimetric changes present in samples containing wildtype template, there is no evidence of amplification as shown by gel electrophoresis.
(TIF)

S5 Fig Peptide nucleic acid suppresses amplification of wildtype IDH1 at copy numbers below 5.0X10^5^. Samples 1–14 contain increasing copy numbers ranging from 6.0X10^4^ to 1.0X10^6^ wildtype synthetic DNA. Samples above 6.0X105 were positive both colorimetrically and electrophoretically. Importantly, all samples without a color change showed no evidence of amplification via gel electrophoresis. These results are representative of two experiments.
(TIF)

S6 Fig Patient-derived tumor lysates processed with 0.4 M NaOH. Alkaline tissue digest was performed for 5 minutes, and lysates were subsequently diluted 1:100 then added to each LAMP reaction at either 2.5 uL or 5.0 uL. Samples 1 and 3 utilize patient sample number 1 (wildtype, Table 1) while samples 2 and 4 utilize patient sample number 3 (mutant, Table 1).
(TIF)

S1 Raw images (PDF)
