# Supplementary figures and images for: Rapid extraction-free detection of the R132H isocitrate dehydrogenase mutation in glioma using colorimetric peptide nucleic acid-loop mediated isothermal amplification (CPNA-LAMP)
Source: PLoS One. 2023 Sep 21;18(9):e0291666. doi: 10.1371/journal.pone.0291666 (PMC10513201; doi:10.1371/journal.pone.0291666)

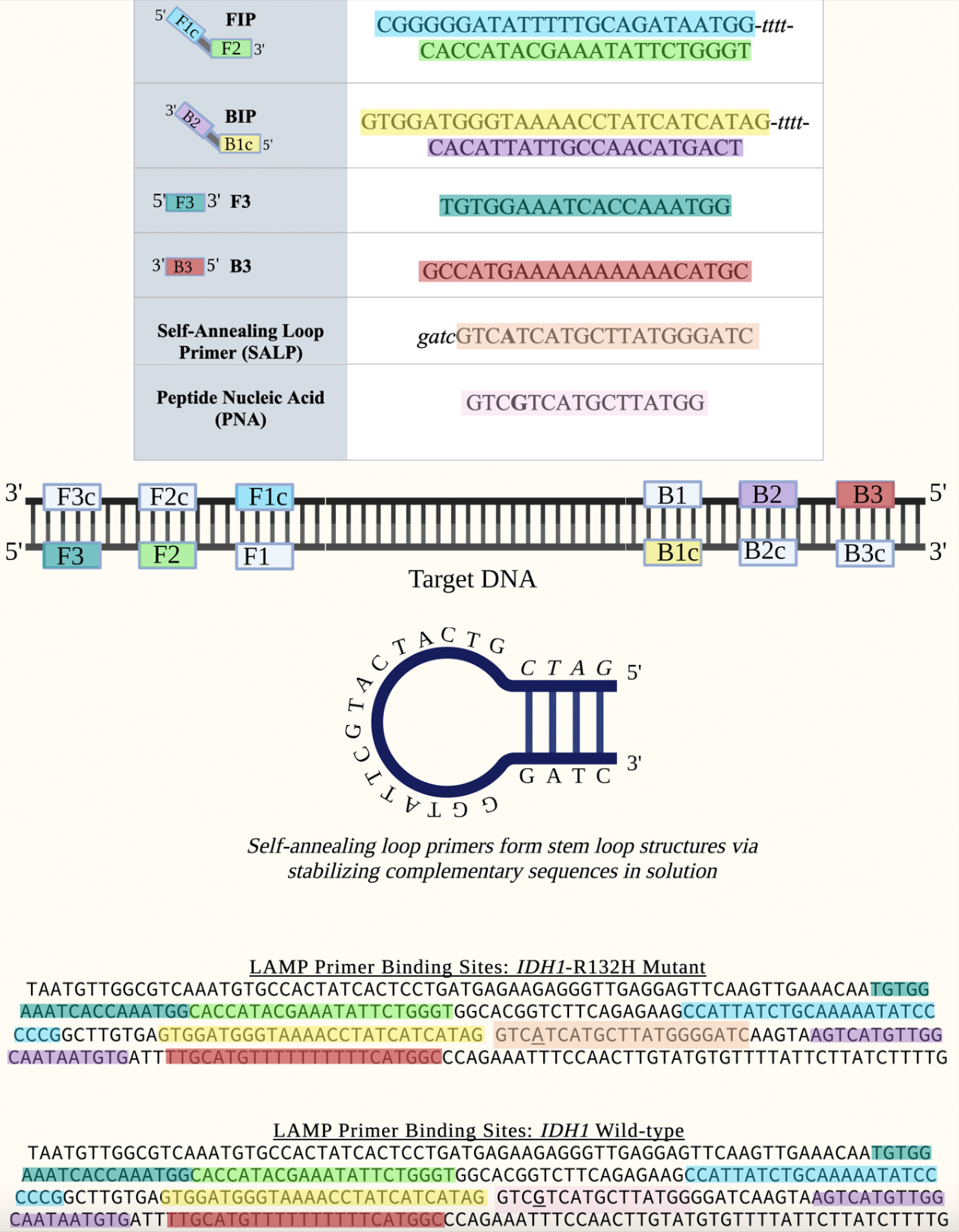

Supplement: S1 Fig — Forward inner primer (FIP) and backward inner primer (BIP) contain two target sequences specific to two different regions in the template DNA. Four thymine linkers provide flexibility for FIP to create secondary structures. The optional addition of loop primers that target dumbbell loops can further expedite amplification. The SALP contains self-complementary ends which form stem-loop structures until bound to the target sequence. (TIF) [file pone.0291666.s001.tif]

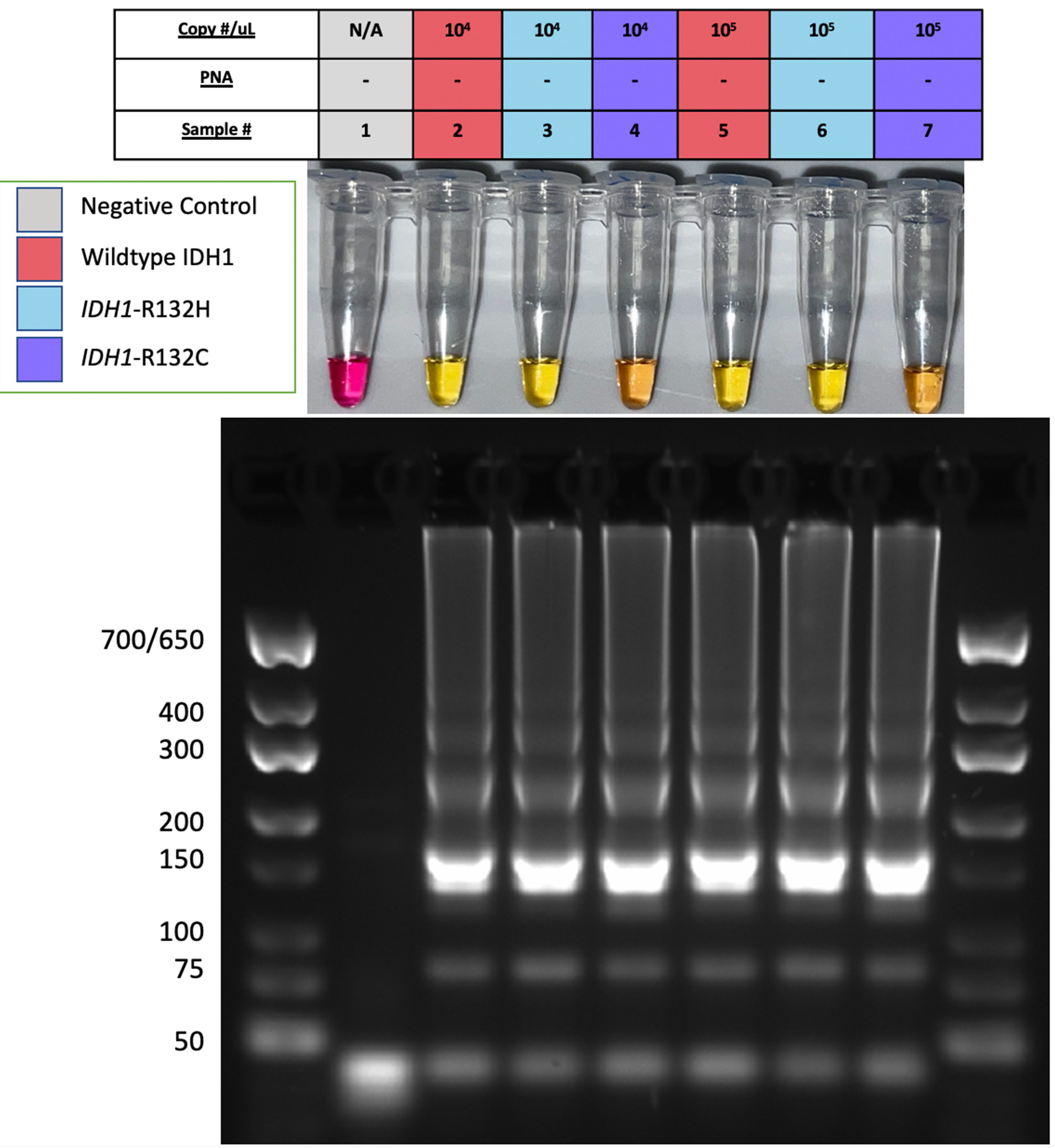

Supplement: S2 Fig — (1) Non-template control, (2) WT at 5.0X104 copies, (3) IDH1-R132H at 5.0X104 copies, (4) IDH1-R132C at 5.0X104 copies, (5) WT at 5.0X105 copies, (6) IDH1-R132H at 5.0X105 copies, (4) IDH1-R132C at 5.0X105 copies. (TIF) [file pone.0291666.s002.tif]

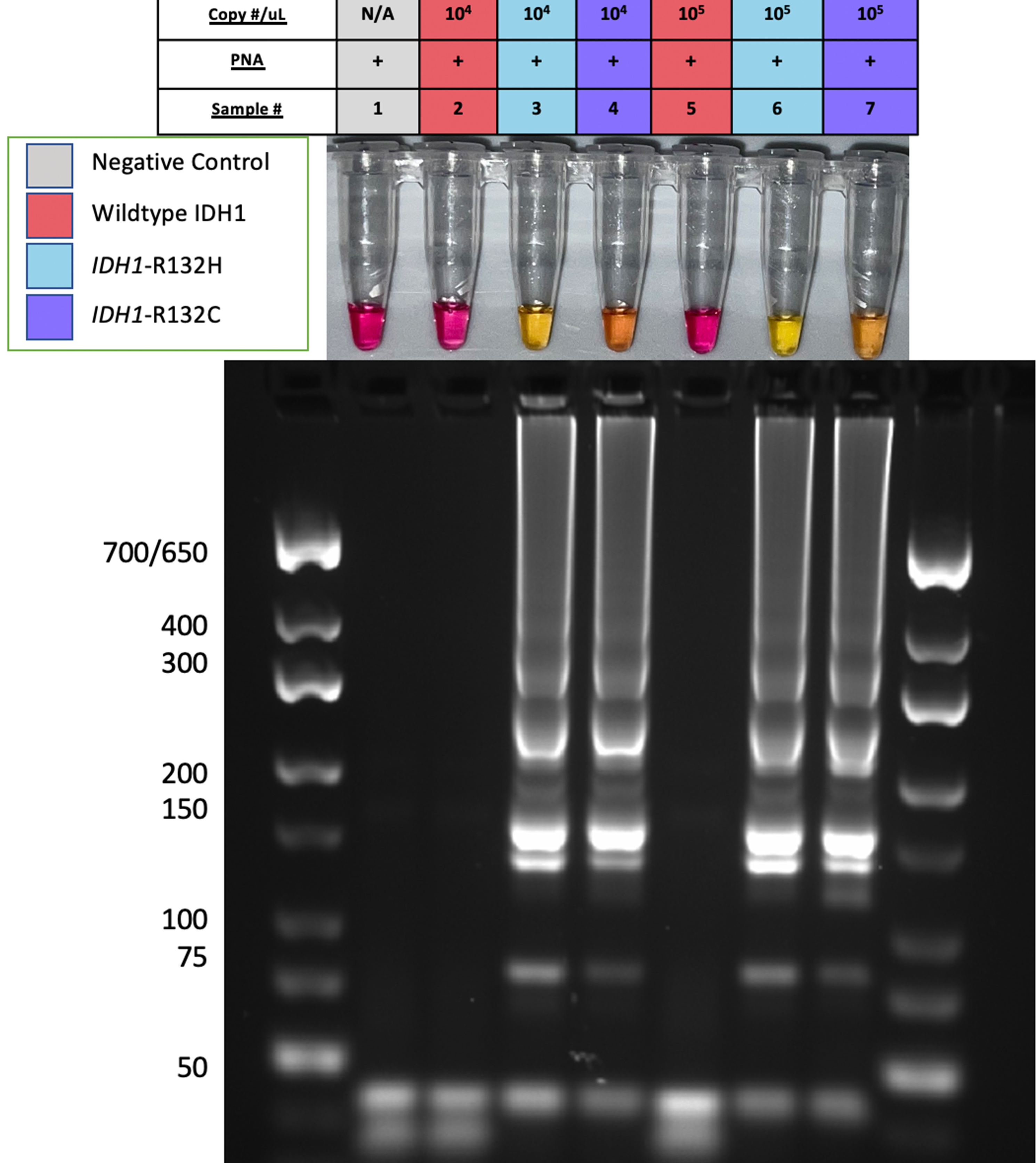

Supplement: S3 Fig — (1) Non-template control, (2) WT at 5.0X104 copies, (3) IDH1-R132H at 5.0X104 copies, (4) IDH1-R132C at 5.0X104 copies, (5) WT at 5.0X105 copies, (6) IDH1-R132H at 5.0X105 copies, (4) IDH1-R132C at 5.0X105 copies. (TIF) [file pone.0291666.s003.tif]

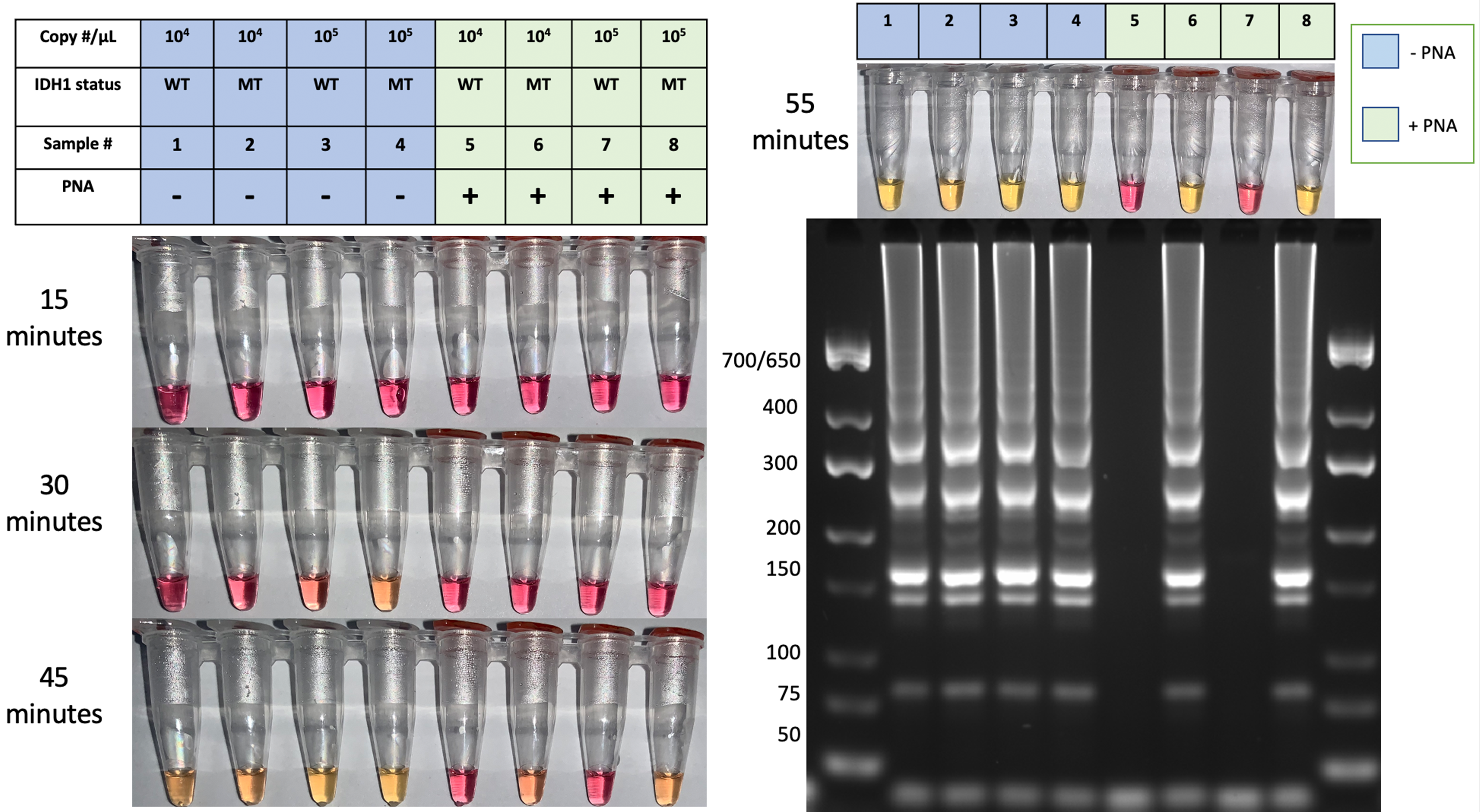

Supplement: S4 Fig — Lanes 1–4 do not contain PNA while lanes 5–8 contain PNA. Odd samples contain IDH1 wildtype synthetic DNA while evenly numbered samples contain IDH1-R132H mutant synthetic DNA. The presence of PNA in the reaction delays colorimetric changes by approximately 10 minutes. Positive results become visually interpretable at 45 minutes, then unambiguous by 55. Higher copy number of DNA results in an earlier and more vibrant colorimetric change. Importantly, at 55 minutes, when there is no colorimetric changes present in samples containing wildtype template, there is no evidence of amplification as shown by gel electrophoresis. (TIF) [file pone.0291666.s004.tif]

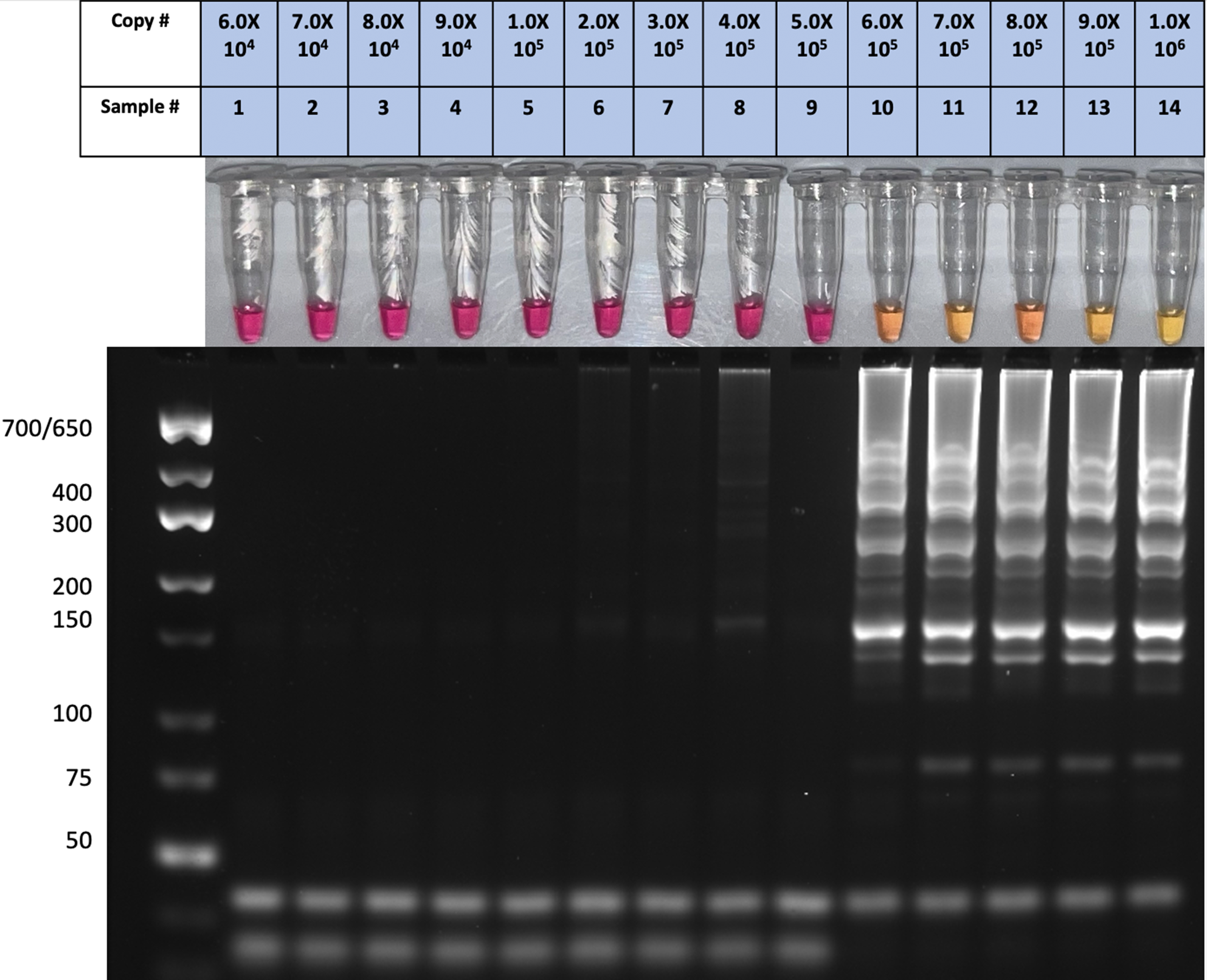

Supplement: S5 Fig — Samples 1–14 contain increasing copy numbers ranging from 6.0X104 to 1.0X106 wildtype synthetic DNA. Samples above 6.0X105 were positive both colorimetrically and electrophoretically. Importantly, all samples without a color change showed no evidence of amplification via gel electrophoresis. These results are representative of two experiments. (TIF) [file pone.0291666.s005.tif]

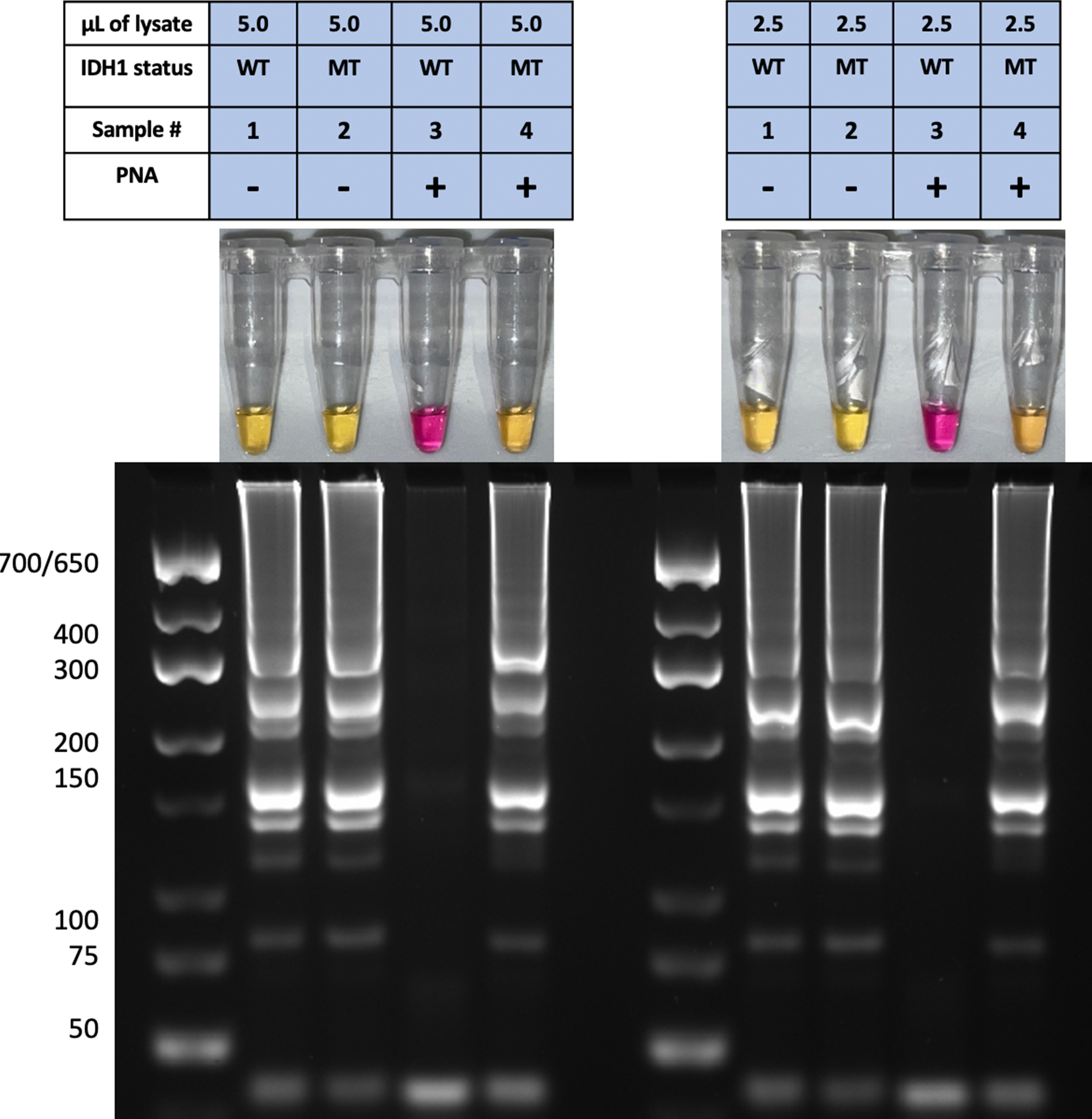

Supplement: S6 Fig — Alkaline tissue digest was performed for 5 minutes, and lysates were subsequently diluted 1:100 then added to each LAMP reaction at either 2.5 uL or 5.0 uL. Samples 1 and 3 utilize patient sample number 1 (wildtype, Table 1) while samples 2 and 4 utilize patient sample number 3 (mutant, Table 1). (TIF) [file pone.0291666.s006.tif]
