# Supplementary material for: Rapid extraction-free detection of the R132H isocitrate dehydrogenase mutation in glioma using colorimetric peptide nucleic acid-loop mediated isothermal amplification (CPNA-LAMP)
Source: PLoS One. 2023 Sep 21;18(9):e0291666. doi: 10.1371/journal.pone.0291666 (PMC10513201; doi:10.1371/journal.pone.0291666)
Supplement: S1 Raw images — (PDF) [file pone.0291666.s007.pdf]

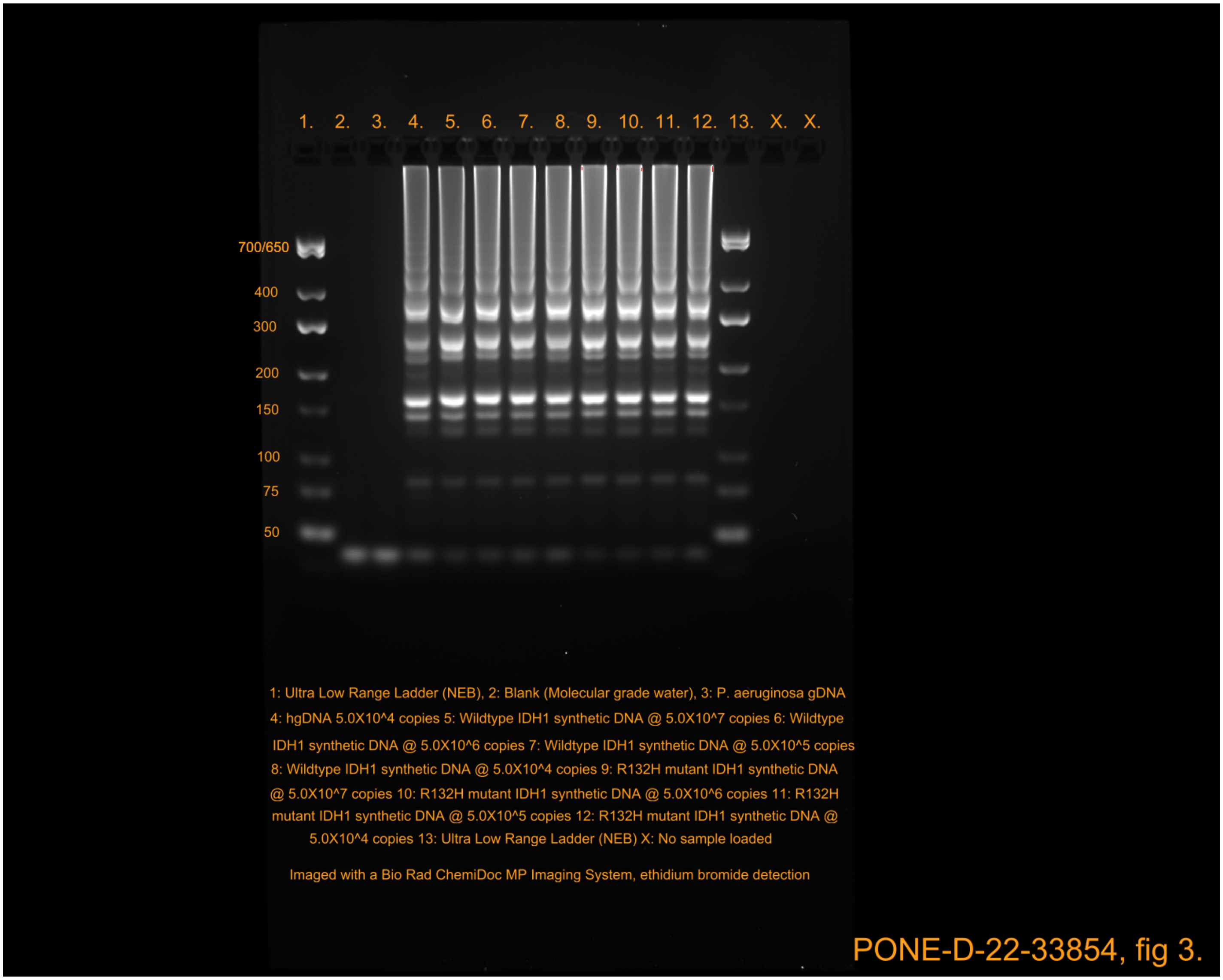
1. 2. 3. 4. 5. 6. 7. 8. 9. 10. 11. 12. 13. X. X.
700/650
400
300
200
150
100
75
50
1: Ultra Low Range Ladder (NEB), 2: Blank (Molecular grade water), 3: P. aeruginosa gDNA
4: hgDNA 5.0X10^4 copies 5: Wildtype IDH1 synthetic DNA @ 5.0X10^7 copies 6: Wildtype
IDH1 synthetic DNA @ 5.0X10^6 copies 7: Wildtype IDH1 synthetic DNA @ 5.0X10^5 copies
8: Wildtype IDH1 synthetic DNA @ 5.0X10^4 copies 9: R132H mutant IDH1 synthetic DNA
@ 5.0X10^7 copies 10: R132H mutant IDH1 synthetic DNA @ 5.0X10^6 copies 11: R132H
mutant IDH1 synthetic DNA @ 5.0X10^5 copies 12: R132H mutant IDH1 synthetic DNA @
5.0X10^4 copies 13: Ultra Low Range Ladder (NEB) X: No sample loaded
Imaged with a Bio Rad ChemiDoc MP Imaging System, ethidium bromide detection
PONE-D-22-33854, fig 3.

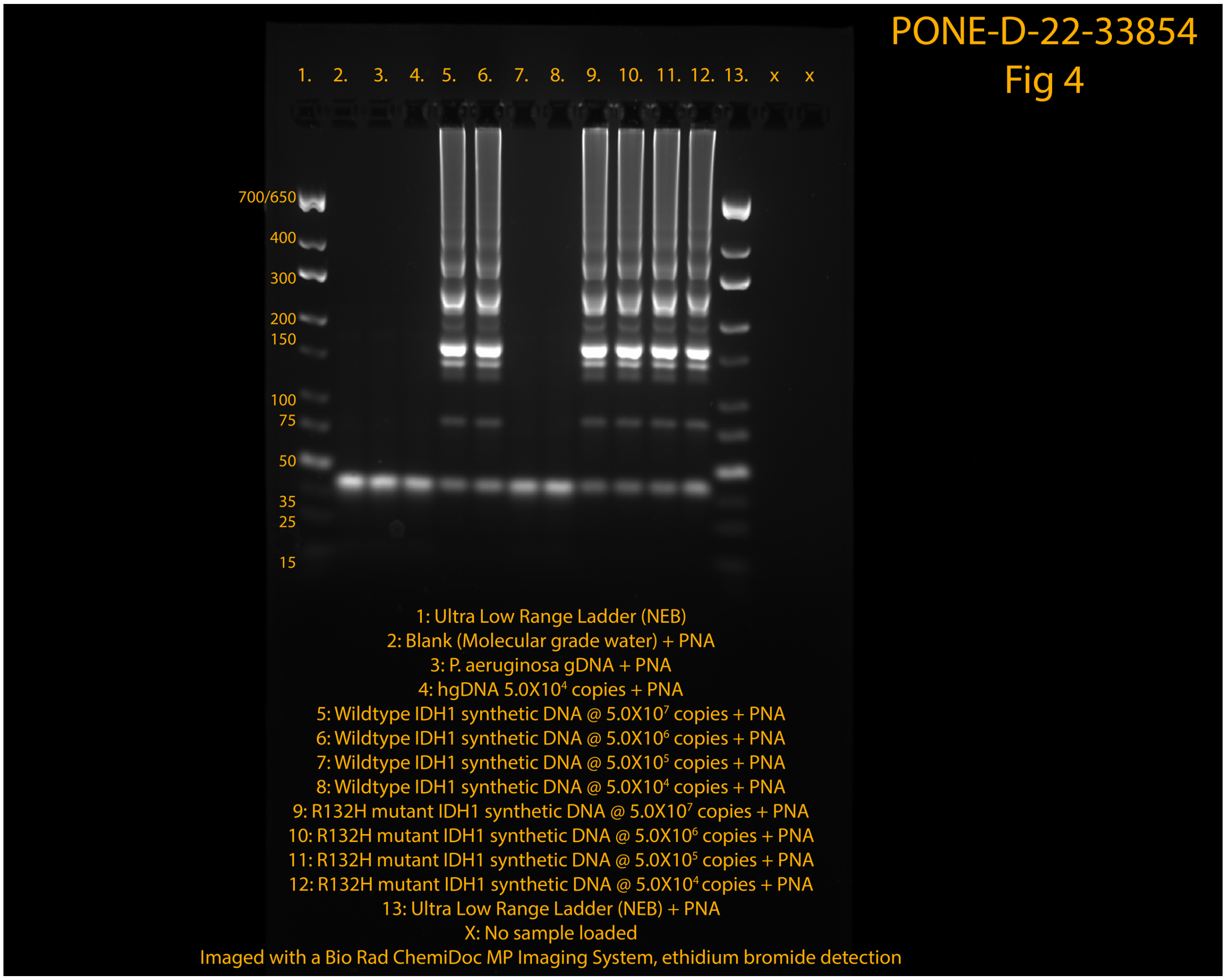
PONE-D-22-33854
Fig 4
1. 2. 3. 4. 5. 6. 7. 8. 9. 10. 11. 12. 13. x x
700/650
400
300
200
150
100
75
50
35
25
15
1: Ultra Low Range Ladder (NEB)
2: Blank (Molecular grade water) + PNA
3: P. aeruginosa gDNA + PNA
4: hgDNA $5.0X10^{4}$ copies + PNA
5: Wildtype IDH1 synthetic DNA @ $5.0X10^{7}$ copies + PNA
6: Wildtype IDH1 synthetic DNA @ $5.0X10^{6}$ copies + PNA
7: Wildtype IDH1 synthetic DNA @ $5.0X10^{5}$ copies + PNA
8: Wildtype IDH1 synthetic DNA @ $5.0X10^{4}$ copies + PNA
9: R132H mutant IDH1 synthetic DNA @ $5.0X10^{7}$ copies + PNA
10: R132H mutant IDH1 synthetic DNA @ $5.0X10^{6}$ copies + PNA
11: R132H mutant IDH1 synthetic DNA @ $5.0X10^{5}$ copies + PNA
12: R132H mutant IDH1 synthetic DNA @ $5.0X10^{4}$ copies + PNA
13: Ultra Low Range Ladder (NEB) + PNA
X: No sample loaded
Imaged with a Bio Rad ChemiDoc MP Imaging System, ethidium bromide detection

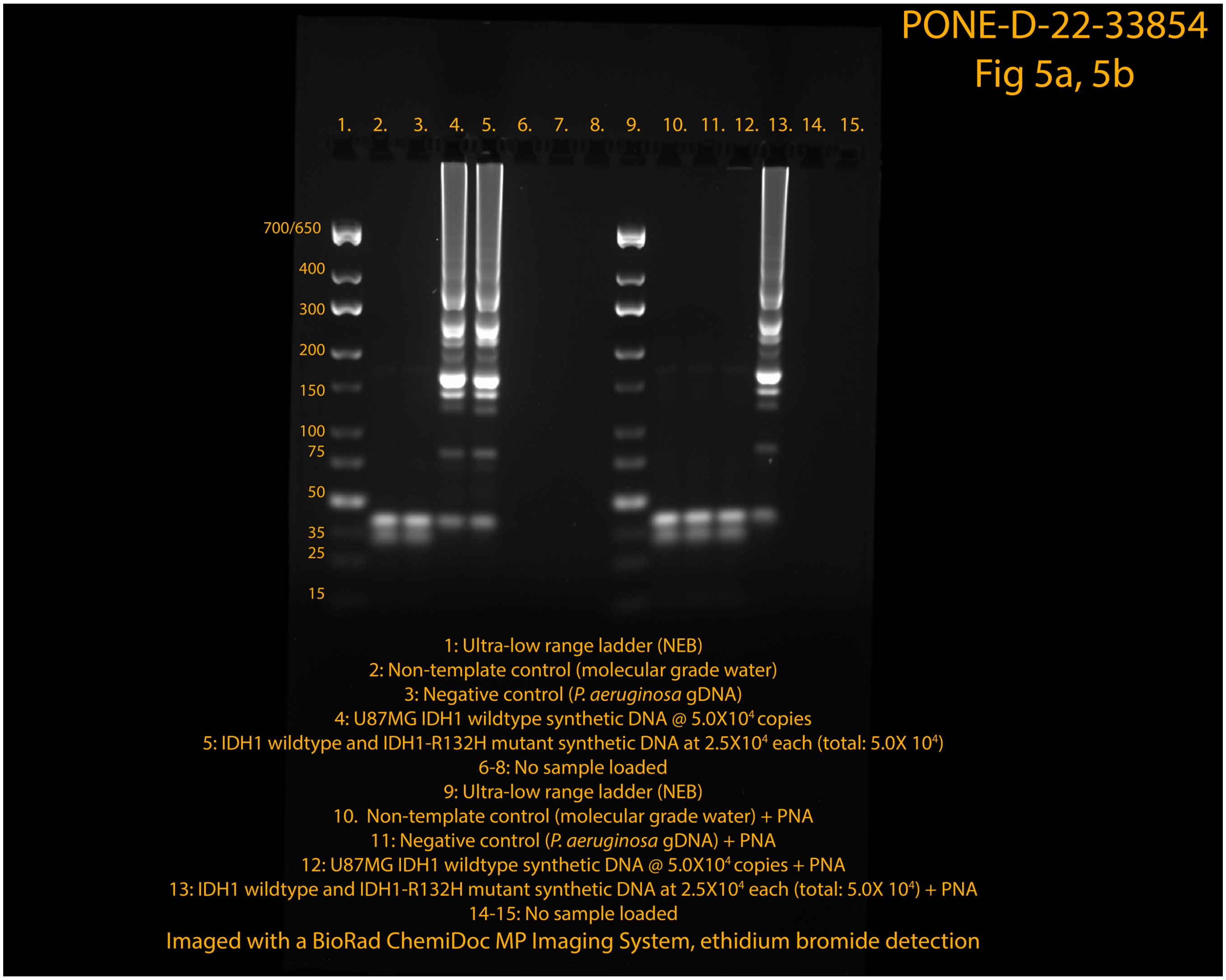
PONE-D-22-33854
Fig 5a, 5b
1. 2. 3. 4. 5. 6. 7. 8. 9. 10. 11. 12. 13. 14. 15.
700/650
400
300
200
150
100
75
50
35
25
15
1: Ultra-low range ladder (NEB)
2: Non-template control (molecular grade water)
3: Negative control (*P. aeruginosa* gDNA)
4: U87MG IDH1 wildtype synthetic DNA @ 5.0X10^4 copies
5: IDH1 wildtype and IDH1-R132H mutant synthetic DNA at 2.5X10^4 each (total: 5.0X 10^4)
6-8: No sample loaded
9: Ultra-low range ladder (NEB)
10. Non-template control (molecular grade water) + PNA
11: Negative control (*P. aeruginosa* gDNA) + PNA
12: U87MG IDH1 wildtype synthetic DNA @ 5.0X10^4 copies + PNA
13: IDH1 wildtype and IDH1-R132H mutant synthetic DNA at 2.5X10^4 each (total: 5.0X 10^4) + PNA
14-15: No sample loaded
Imaged with a BioRad ChemiDoc MP Imaging System, ethidium bromide detection

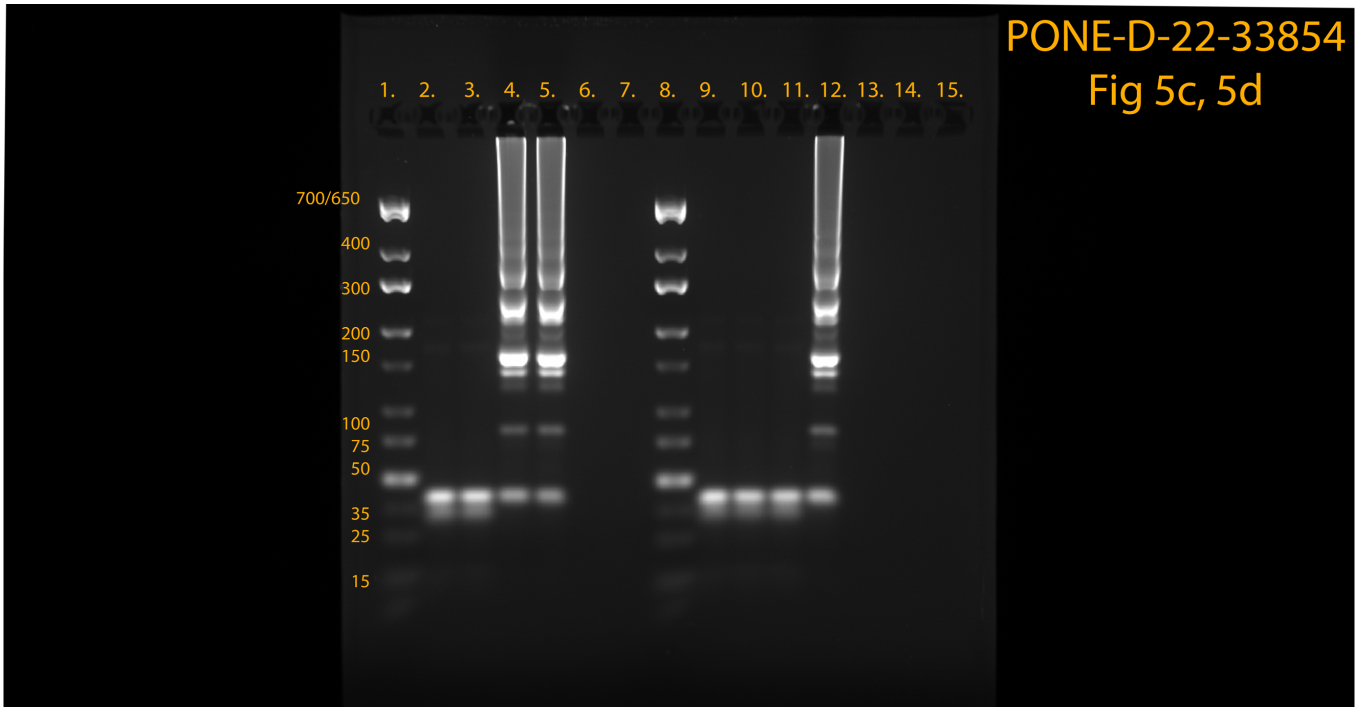

1: Ultra-low range ladder (NEB)
2: Non-template control (molecular grade water)
3: Negative control (*P. aeruginosa* gDNA)
4: U87MG IDH1 R132H mutant synthetic DNA @ 5.0X10^4 copies
5: IDH1 wildtype purified human genomic DNA and IDH1-R132H mutant synthetic DNA at 2.5X10^4 each (total: 5.0X 10^4)
6-7: No sample loaded
8: Ultra-low range ladder (NEB)
9. Non-template control (molecular grade water) + PNA
10: Negative control (*P. aeruginosa* gDNA) + PNA
11: Purified human genomic DNA @ 5.0X10^4 copies + PNA
12: purified human genomic DNA (IDH1 wildtype) and IDH1-R132H mutant synthetic DNA at 2.5X10^4 each (total: 5.0X 10^4) + PNA
13-15: No sample loaded
Imaged with a BioRad ChemiDoc MP Imaging System, ethidium bromide detection

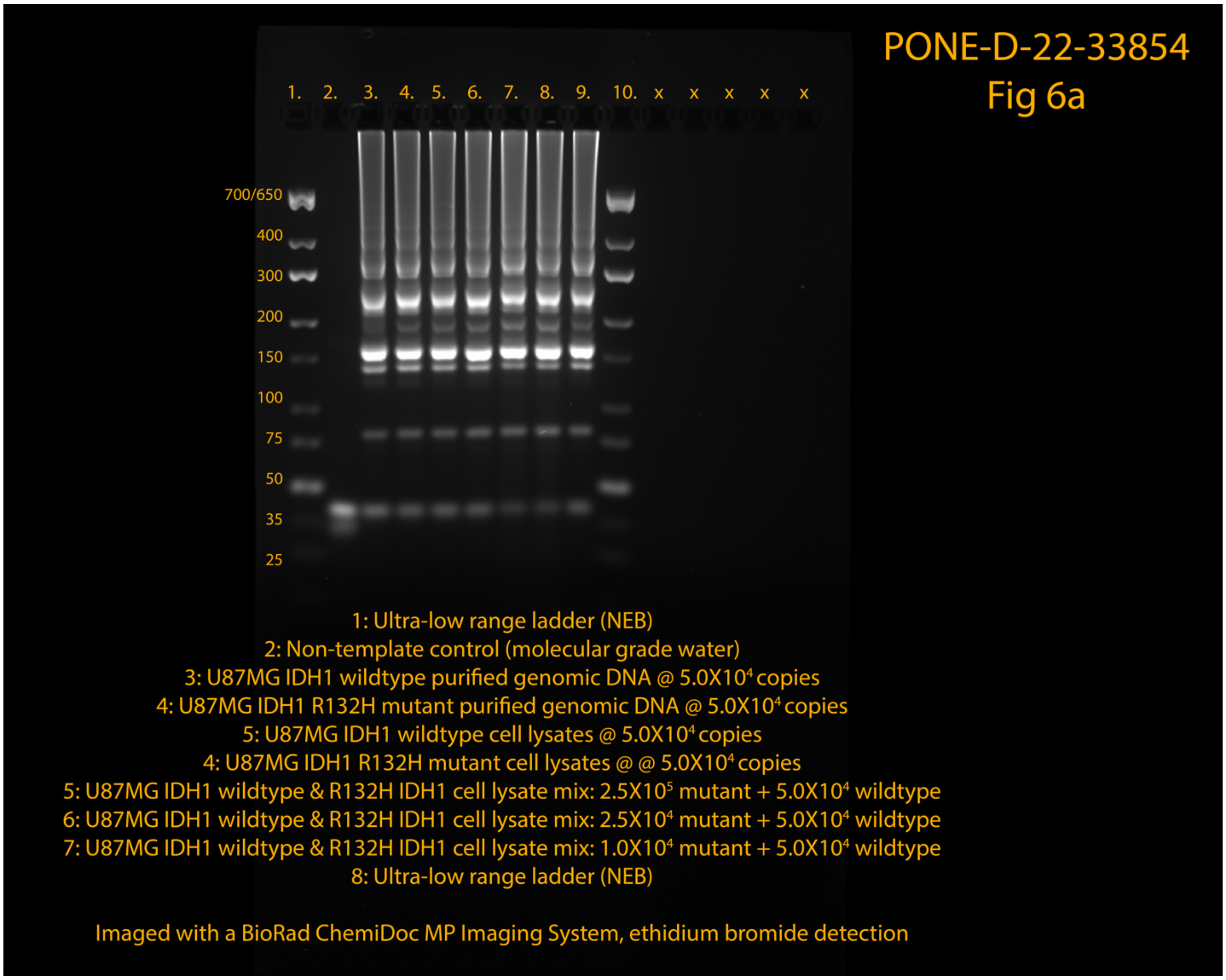
PONE-D-22-33854
Fig 6a
1. 2. 3. 4. 5. 6. 7. 8. 9. 10. x x x x x
700/650
400
300
200
150
100
75
50
35
25
1: Ultra-low range ladder (NEB)
2: Non-template control (molecular grade water)
3: U87MG IDH1 wildtype purified genomic DNA @ 5.0X10^{4} copies
4: U87MG IDH1 R132H mutant purified genomic DNA @ 5.0X10^{4} copies
5: U87MG IDH1 wildtype cell lysates @ 5.0X10^{4} copies
4: U87MG IDH1 R132H mutant cell lysates @ @ 5.0X10^{4} copies
5: U87MG IDH1 wildtype & R132H IDH1 cell lysate mix: 2.5X10^{5} mutant + 5.0X10^{4} wildtype
6: U87MG IDH1 wildtype & R132H IDH1 cell lysate mix: 2.5X10^{4} mutant + 5.0X10^{4} wildtype
7: U87MG IDH1 wildtype & R132H IDH1 cell lysate mix: 1.0X10^{4} mutant + 5.0X10^{4} wildtype
8: Ultra-low range ladder (NEB)
Imaged with a BioRad ChemiDoc MP Imaging System, ethidium bromide detection

PONE-D-22-33854
fig 6b

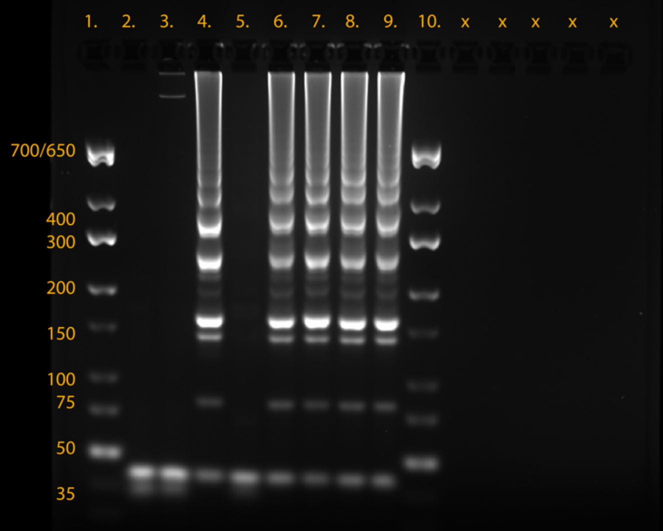

1: Ultra-low range ladder (NEB)
2: Non-template control (molecular grade water)
3: U87MG IDH1 wildtype purified genomic DNA @ $5.0X10^{4}$ copies + PNA
4: U87MG IDH1 R132H mutant purified genomic DNA @ $5.0X10^{4}$ copies + PNA
5: U87MG IDH1 wildtype cell lysates @ $5.0X10^{4}$ copies + PNA
4: U87MG IDH1 R132H mutant cell lysates @ @ $5.0X10^{4}$ copies + PNA
5: U87MG IDH1 wildtype & R132H IDH1 cell lysate mix: $2.5X10^{5}$ mutant + $5.0X10^{4}$ wildtype + PNA
6: U87MG IDH1 wildtype & R132H IDH1 cell lysate mix: $2.5X10^{4}$ mutant + $5.0X10^{4}$ wildtype + PNA
7: U87MG IDH1 wildtype & R132H IDH1 cell lysate mix: $1.0X10^{4}$ mutant + $5.0X10^{4}$ wildtype + PNA
8: Ultra-low range ladder (NEB)

Imaged with a BioRad ChemiDoc MP Imaging System, ethidium bromide detection

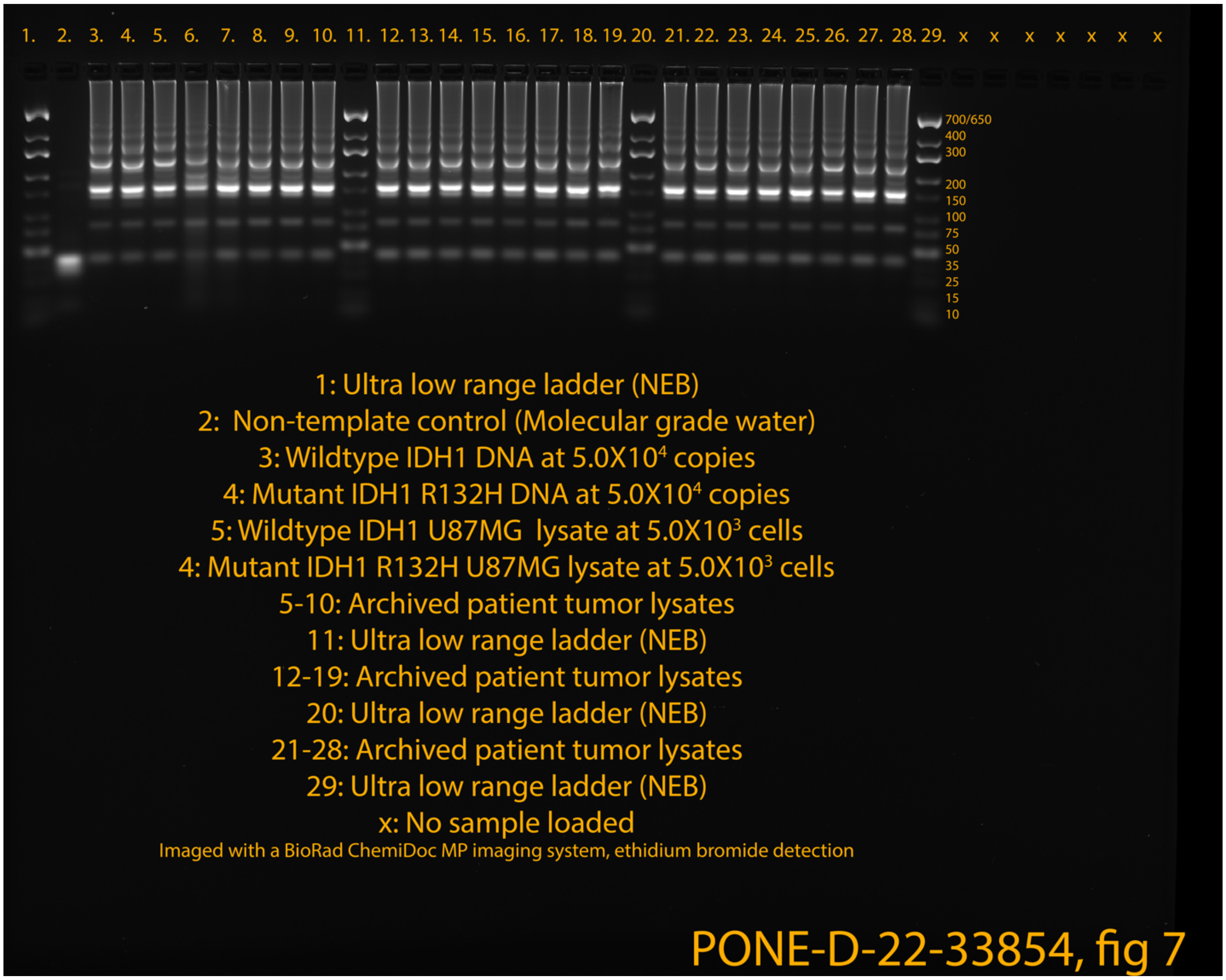
1. 2. 3. 4. 5. 6. 7. 8. 9. 10. 11. 12. 13. 14. 15. 16. 17. 18. 19. 20. 21. 22. 23. 24. 25. 26. 27. 28. 29. x x x x x x x
700/650
400
300
200
150
100
75
50
35
25
15
10
1: Ultra low range ladder (NEB)
2: Non-template control (Molecular grade water)
3: Wildtype IDH1 DNA at 5.0X10^4 copies
4: Mutant IDH1 R132H DNA at 5.0X10^4 copies
5: Wildtype IDH1 U87MG lysate at 5.0X10^3 cells
4: Mutant IDH1 R132H U87MG lysate at 5.0X10^3 cells
5-10: Archived patient tumor lysates
11: Ultra low range ladder (NEB)
12-19: Archived patient tumor lysates
20: Ultra low range ladder (NEB)
21-28: Archived patient tumor lysates
29: Ultra low range ladder (NEB)
x: No sample loaded
Imaged with a BioRad ChemiDoc MP imaging system, ethidium bromide detection
PONE-D-22-33854, fig 7

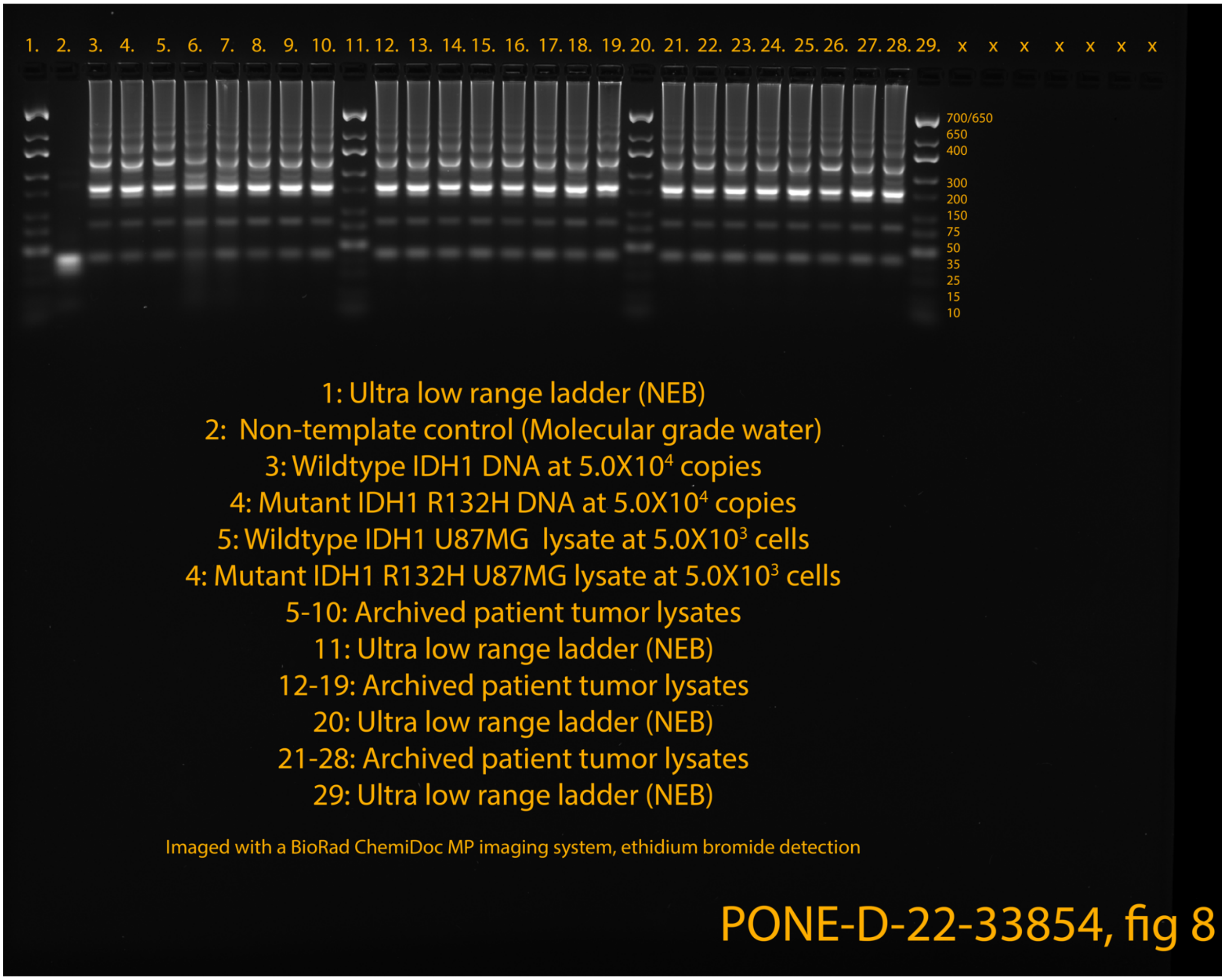
1. 2. 3. 4. 5. 6. 7. 8. 9. 10. 11. 12. 13. 14. 15. 16. 17. 18. 19. 20. 21. 22. 23. 24. 25. 26. 27. 28. 29. x x x x x x x
700/650
650
400
300
200
150
75
50
35
25
15
10
1: Ultra low range ladder (NEB)
2: Non-template control (Molecular grade water)
3: Wildtype IDH1 DNA at 5.0X10^4 copies
4: Mutant IDH1 R132H DNA at 5.0X10^4 copies
5: Wildtype IDH1 U87MG lysate at 5.0X10^3 cells
4: Mutant IDH1 R132H U87MG lysate at 5.0X10^3 cells
5-10: Archived patient tumor lysates
11: Ultra low range ladder (NEB)
12-19: Archived patient tumor lysates
20: Ultra low range ladder (NEB)
21-28: Archived patient tumor lysates
29: Ultra low range ladder (NEB)
Imaged with a BioRad ChemiDoc MP imaging system, ethidium bromide detection
PONE-D-22-33854, fig 8

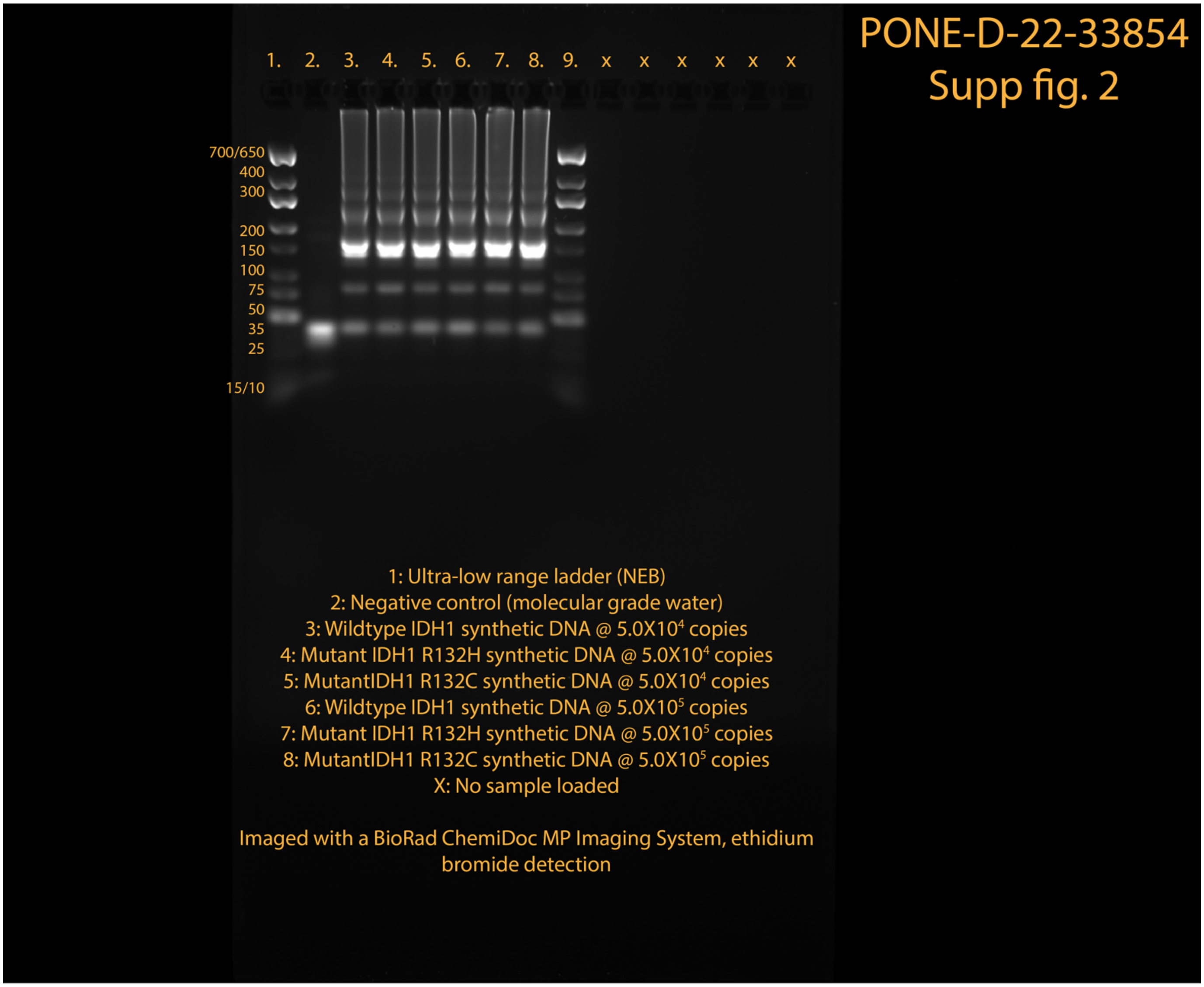
PONE-D-22-33854
Supp fig. 2
1. 2. 3. 4. 5. 6. 7. 8. 9. x x x x x x
700/650
400
300
200
150
100
75
50
35
25
15/10
1: Ultra-low range ladder (NEB)
2: Negative control (molecular grade water)
3: Wildtype IDH1 synthetic DNA @ 5.0X10^{4} copies
4: Mutant IDH1 R132H synthetic DNA @ 5.0X10^{4} copies
5: MutantIDH1 R132C synthetic DNA @ 5.0X10^{4} copies
6: Wildtype IDH1 synthetic DNA @ 5.0X10^{5} copies
7: Mutant IDH1 R132H synthetic DNA @ 5.0X10^{5} copies
8: MutantIDH1 R132C synthetic DNA @ 5.0X10^{5} copies
X: No sample loaded
Imaged with a BioRad ChemiDoc MP Imaging System, ethidium bromide detection

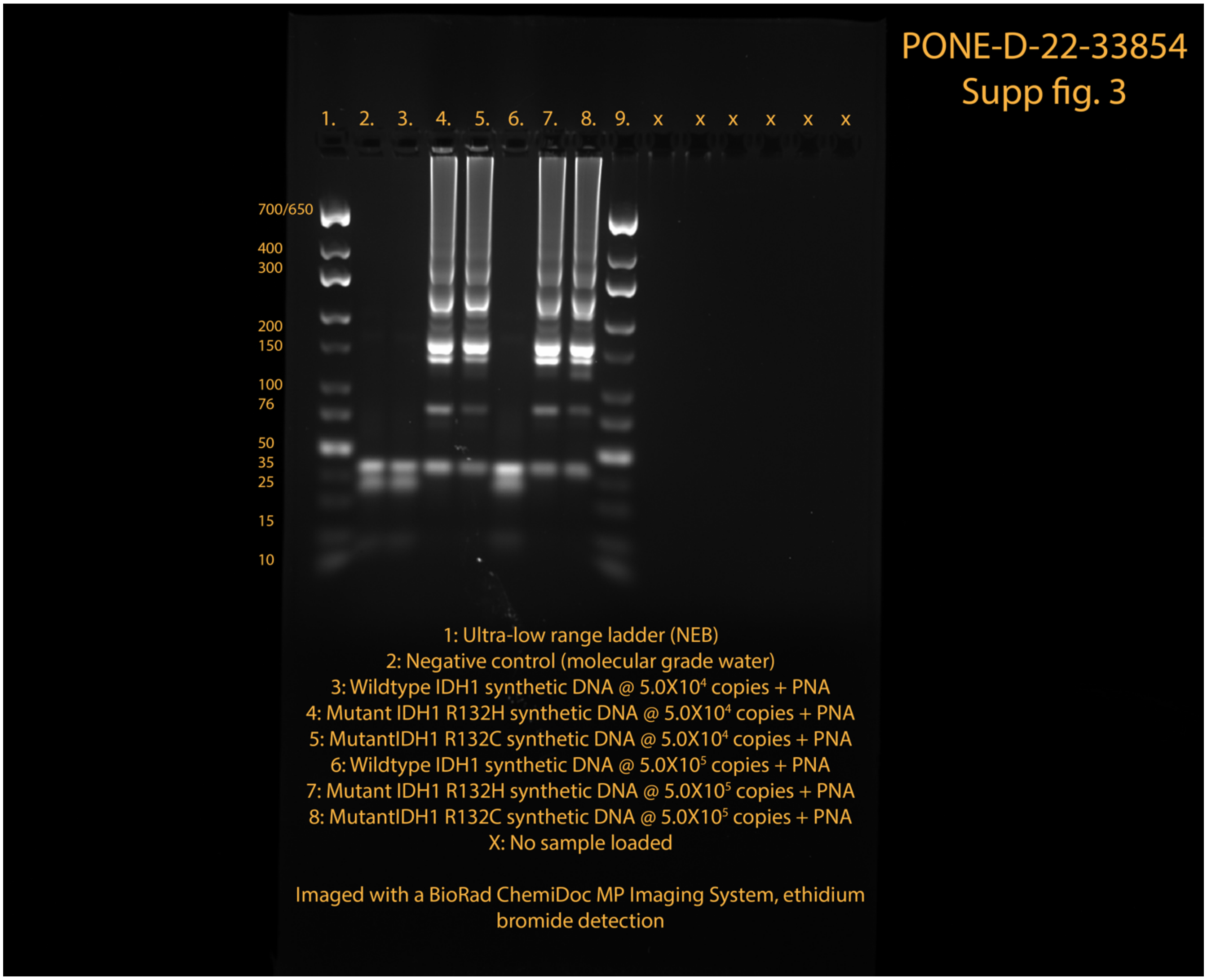
PONE-D-22-33854
Supp fig. 3
1. 2. 3. 4. 5. 6. 7. 8. 9. x x x x x x
700/650
400
300
200
150
100
76
50
35
25
15
10
1: Ultra-low range ladder (NEB)
2: Negative control (molecular grade water)
3: Wildtype IDH1 synthetic DNA @ 5.0X10^4 copies + PNA
4: Mutant IDH1 R132H synthetic DNA @ 5.0X10^4 copies + PNA
5: MutantIDH1 R132C synthetic DNA @ 5.0X10^4 copies + PNA
6: Wildtype IDH1 synthetic DNA @ 5.0X10^5 copies + PNA
7: Mutant IDH1 R132H synthetic DNA @ 5.0X10^5 copies + PNA
8: MutantIDH1 R132C synthetic DNA @ 5.0X10^5 copies + PNA
X: No sample loaded
Imaged with a BioRad ChemiDoc MP Imaging System, ethidium bromide detection

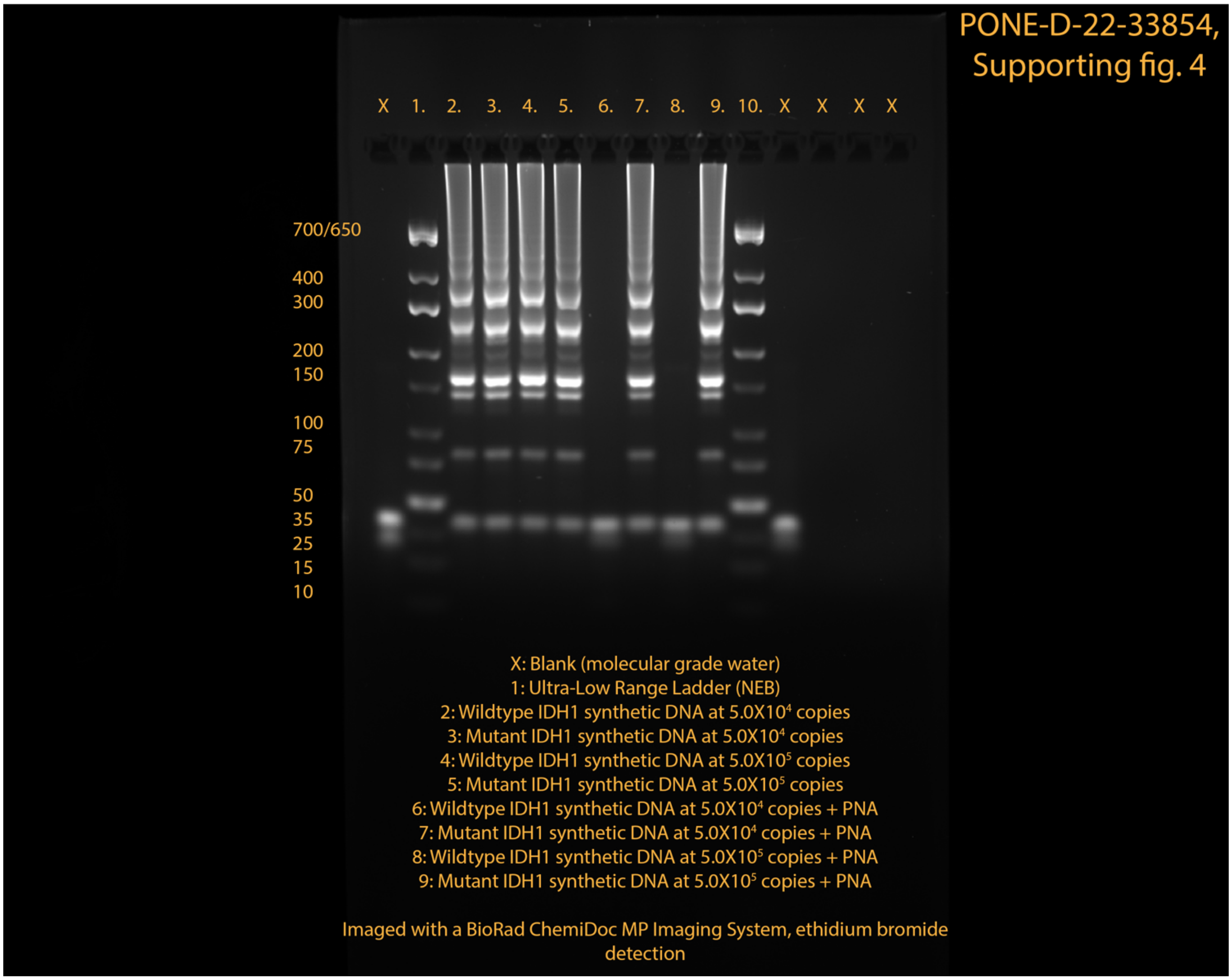
PONE-D-22-33854, Supporting fig. 4
X 1. 2. 3. 4. 5. 6. 7. 8. 9. 10. X X X X
700/650
400
300
200
150
100
75
50
35
25
15
10
X: Blank (molecular grade water)
1: Ultra-Low Range Ladder (NEB)
2: Wildtype IDH1 synthetic DNA at $5.0X10^4$ copies
3: Mutant IDH1 synthetic DNA at $5.0X10^4$ copies
4: Wildtype IDH1 synthetic DNA at $5.0X10^5$ copies
5: Mutant IDH1 synthetic DNA at $5.0X10^5$ copies
6: Wildtype IDH1 synthetic DNA at $5.0X10^4$ copies + PNA
7: Mutant IDH1 synthetic DNA at $5.0X10^4$ copies + PNA
8: Wildtype IDH1 synthetic DNA at $5.0X10^5$ copies + PNA
9: Mutant IDH1 synthetic DNA at $5.0X10^5$ copies + PNA
Imaged with a BioRad ChemiDoc MP Imaging System, ethidium bromide detection

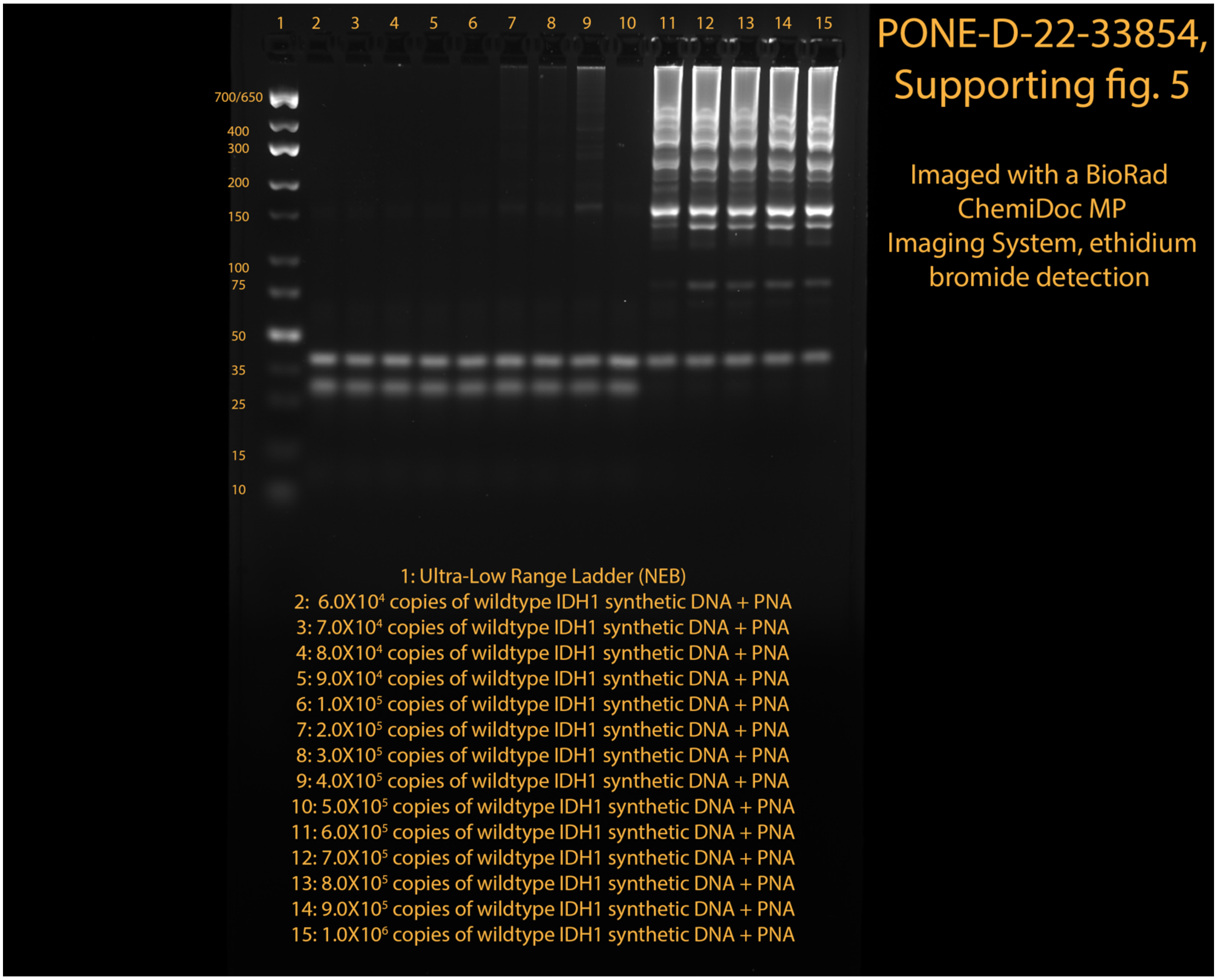
PONE-D-22-33854, Supporting fig. 5
Imaged with a BioRad ChemiDoc MP Imaging System, ethidium bromide detection
1 2 3 4 5 6 7 8 9 10 11 12 13 14 15
700/650
400
300
200
150
100
75
50
35
25
15
10
1: Ultra-Low Range Ladder (NEB)
2: 6.0X10^4 copies of wildtype IDH1 synthetic DNA + PNA
3: 7.0X10^4 copies of wildtype IDH1 synthetic DNA + PNA
4: 8.0X10^4 copies of wildtype IDH1 synthetic DNA + PNA
5: 9.0X10^4 copies of wildtype IDH1 synthetic DNA + PNA
6: 1.0X10^5 copies of wildtype IDH1 synthetic DNA + PNA
7: 2.0X10^5 copies of wildtype IDH1 synthetic DNA + PNA
8: 3.0X10^5 copies of wildtype IDH1 synthetic DNA + PNA
9: 4.0X10^5 copies of wildtype IDH1 synthetic DNA + PNA
10: 5.0X10^5 copies of wildtype IDH1 synthetic DNA + PNA
11: 6.0X10^5 copies of wildtype IDH1 synthetic DNA + PNA
12: 7.0X10^5 copies of wildtype IDH1 synthetic DNA + PNA
13: 8.0X10^5 copies of wildtype IDH1 synthetic DNA + PNA
14: 9.0X10^5 copies of wildtype IDH1 synthetic DNA + PNA
15: 1.0X10^6 copies of wildtype IDH1 synthetic DNA + PNA

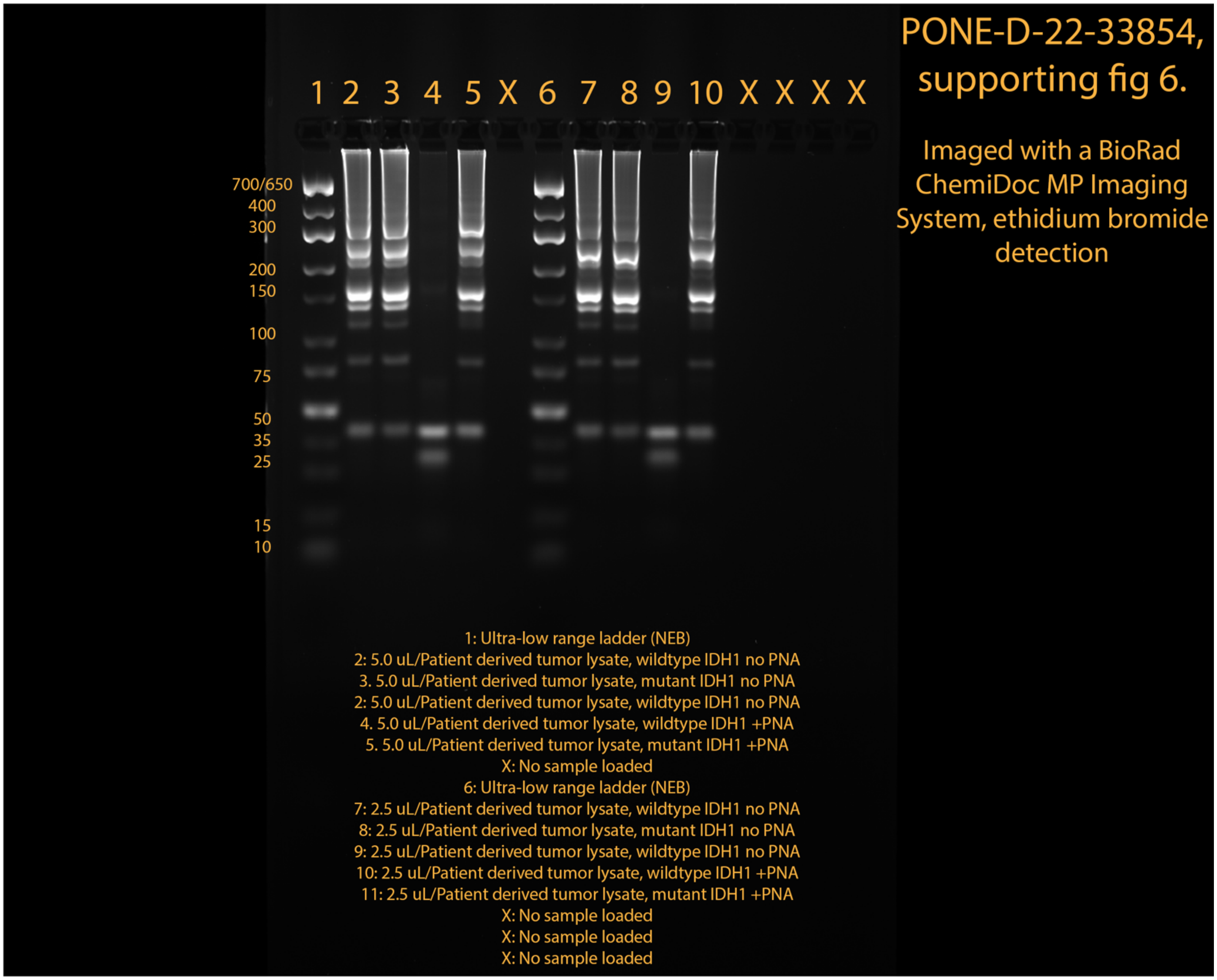
PONE-D-22-33854, supporting fig 6.
Imaged with a BioRad ChemiDoc MP Imaging System, ethidium bromide detection
1 2 3 4 5 X 6 7 8 9 10 X X X X
700/650
400
300
200
150
100
75
50
35
25
15
10
1: Ultra-low range ladder (NEB)
2: 5.0 uL/Patient derived tumor lysate, wildtype IDH1 no PNA
3. 5.0 uL/Patient derived tumor lysate, mutant IDH1 no PNA
2: 5.0 uL/Patient derived tumor lysate, wildtype IDH1 no PNA
4. 5.0 uL/Patient derived tumor lysate, wildtype IDH1 +PNA
5. 5.0 uL/Patient derived tumor lysate, mutant IDH1 +PNA
X: No sample loaded
6: Ultra-low range ladder (NEB)
7: 2.5 uL/Patient derived tumor lysate, wildtype IDH1 no PNA
8: 2.5 uL/Patient derived tumor lysate, mutant IDH1 no PNA
9: 2.5 uL/Patient derived tumor lysate, wildtype IDH1 no PNA
10: 2.5 uL/Patient derived tumor lysate, wildtype IDH1 +PNA
11: 2.5 uL/Patient derived tumor lysate, mutant IDH1 +PNA
X: No sample loaded
X: No sample loaded
X: No sample loaded
